# Sex differences in the real-world use of rimegepant for short-term prevention of migraine in the United States

**DOI:** 10.3389/fneur.2026.1901123

**Published:** 2026-09-24

**Authors:** Teshamae Monteith, Jessica Cirillo, Karina Nakajima, Martine C. Maculaitis, Elizabeth McCaig, Nemin Chen, Lucy Abraham, Meghan Fajardo, Feng Dai, Jessica Ailani

**Affiliations:** 1 University of Miami Health System, Miami, FL, United States; 2 Pfizer, Inc., New York, NY, United States; 3 Oracle Life Sciences, Austin, TX, United States; 4 Pfizer, Ltd., Surrey, United Kingdom; 5 Medstar Georgetown University Hospital, Washington, DC, United States

**Keywords:** migraine, preventive, rimegepant, sex differences, short-term prevention

## Abstract

**Introduction:**

Short-term prevention (STP) is a targeted treatment approach to minimize migraine attacks during specific, time-limited periods. Because migraine disproportionately affects women, this study evaluated sex-related differences in the context of rimegepant for STP.

**Methods:**

A cross-sectional observational study collected real-world data via an online survey. Eligible subjects were US residents aged ≥18 years with self-reported episodic or chronic migraine, receiving rimegepant, initiated ≥3 months, and taken within 30 days of survey completion. Reported *p* values are nominal (unadjusted).

**Results:**

Of 425 adults, 152 used rimegepant for STP (female, *n* = 98 [64.5%]; male, *n* = 54 [35.5%]). Women were more likely than men to use rimegepant for STP due to doctor recommendation (69.4% vs. 48.1%, *p* = 0.014), frequency (43.9% vs. 25.9%, *p* = 0.036), and severity of attacks (43.9% vs. 22.2%, *p* = 0.008). In women and men, stress (women, 74.5%; men, 59.3%), disruptive sleeping patterns (women, 53.1%; men, 35.2%), and noise (women, 48.0%; men, 35.2%) were the most common factors provocative of a migraine attack. Across sexes, ≥73% were “satisfied” to “extremely satisfied” with rimegepant for STP, men reported numerically higher levels of satisfaction than women with taking rimegepant as an STP treatment one time (94.4% [17/18] vs. 73.0% [27/37], *p* = 0.08).

**Conclusion:**

This study highlights how migraine differentially affects women and men. Subtle variations by sex in factors provocative of a migraine attack and reasons for rimegepant use were observed. Regardless of sex, rimegepant has potential for STP of migraine. Women and men reported high levels of satisfaction with rimegepant for STP when taken as needed, every other day, or every day.

## 1 Introduction

Migraine, a chronic neurological disease characterized by recurring attacks of pain, nausea, and sensory sensitivity, is associated with substantial disability, resulting in considerable impairment to quality of life (1, 2). Migraine disproportionately affects women, with a cumulative lifetime incidence of 43% for women and 18% for men (3). Women experience more frequent and severe migraine attacks, a greater number of medical comorbidities, and have a higher use of emergency services and urgent care for headache compared with men (4–6). A large European population-based study of over 60,000 individuals reported that sex differences in migraine prevalence and severity (frequency of attack and pain intensity) stemmed almost exclusively from migraine without aura, with no sex differences among individuals with migraine with aura (7). The prevalence of migraine without aura in men and women was also age dependent, with the highest peak during reproductive years, whereas prevalence was not age dependent for men and women with migraine with aura (7). The average ages of migraine onset and cessation in women with migraine without aura is 27.7 and 50.7 years, respectively (8). The corresponding values for women with migraine with aura are 26.7 and 49.5 years. More women with migraine with aura compared with those with migraine without aura have migraine onset before menarche, but similar proportions experience cessation of migraine before menopause (8). There appears to be no difference in the proportion of women with migraine with aura compared with those with migraine without aura who experience migraine after menopause (8). Although migraine predominantly affects women, the conceptualization of migraine as a “women’s disorder” may have exacerbated sex differences in the diagnosis and treatment of migraine, with men less likely to seek treatment, be diagnosed with migraine, or prescribed migraine treatment (9).

The calcitonin gene-related peptide (CGRP) and its receptor have emerged as a key mediator of migraine and are the target of several therapeutic advances. Sex differences in CGRP are evident and may play role in the differential impact of migraine in men and women. Notably, plasma CGRP levels are higher in women than in men (10). CGRP levels may also be regulated by the sex hormone, estrogen. Because fluctuations in estrogen levels can influence the susceptibility to migraine in women (11), it is important to consider the potential impact of biological sex on CGRP-targeting treatments (10).

Migraine treatment approaches can be acute or preventive: acute treatments are taken during a migraine attack to relieve symptoms, and preventive treatments are taken to reduce the frequency and severity of attacks. Biological sex may influence clinical outcomes of patients with migraine treated with CGRP-targeting therapies. *Post hoc* and pooled data studies have reported no significant differences among male and female subjects receiving preventive treatment with CGRP-targeting therapies (12–16). However, in the acute treatment of migraine, potential differential effects of CGRP-targeting therapies in female and male subjects are evident for 2-h pain freedom and 2-h freedom from most bothersome symptom (17).

In general, migraine preventive treatments are for long-term prevention (LTP) taken on a continuous basis. Certain factors and situations can increase the likelihood of a migraine attack, including fluctuations in sex hormones, stress, poor sleep quality, weather changes, and certain chemical substances, such as alcohol (18). Accordingly, patients who are aware of which factors reliably increase the likelihood of an attack may prefer to limit preventive treatment exposure to these scenarios, rather than receive continuous LTP. An expert consensus, which used a modified Delphi process, agreed on the importance of customizing treatment for people with migraine and the utility of “situational prevention,” or “short-term prevention (STP)” (19). As such, many patients are their own experts and can often identify situations that reliably increase their likelihood of experiencing a migraine. STP treatment of migraine may address patient needs by allowing tailored treatment for predictable situations or specific periods when migraine avoidance is most important to the respective patient. While preventive migraine treatments may be taken preemptively in certain situations, such as for exercise-induced migraine, at present there are no treatments specifically indicated for STP treatment of migraine. Certain acute treatments, including triptans and CGRP-targeted therapies, have shown potential as STP treatments, with further research warranted (20–24). However, established limitations of treatments such as triptans, including medication overuse headache, may complicate their use as a STP treatment (25).

Rimegepant, an orally disintegrating 75 mg tablet, is a CGRP receptor antagonist approved for both acute and preventive treatment of migraine. It is available in several countries, including the United States (US), United Kingdom (UK), and countries throughout Europe (26). Rimegepant is rapidly absorbed with a maximum observed plasma concentration from 1–3.5 h and has a half-life of approximately 11 h, has established efficacy for the acute and preventive treatment of migraine, 48-h sustained efficacy with a single dose, and is generally not associated with medication overuse headache (26, 27). As such, rimegepant may be well-suited to serve the need for STP. It is likely that many patients are currently using rimegepant as a STP treatment (24, 28), however it is not well understood whether there are differences between males and females in their use of rimegepant as an STP treatment. Therefore, this study aimed to gather real-world evidence from subjects with migraine using rimegepant for STP treatment and to explore potential sex-related differences in factors or events provocative of a migraine attack, treatment use, and subject satisfaction.

## 2 Materials and methods

### 2.1 Study design

This was a cross-sectional observational study designed to capture real-world subject experiences with rimegepant, including its use for STP of migraine. Data were collected using an online survey administered in the United States. The study was conducted in 2 phases, with both phases administered by a global research company specializing in data collection^1^ and overseen by Oracle Life Sciences^2^. Phase 1 focused on protocol and survey development and cognitive pre-test interviews. Survey development used a combination of validated scales and custom questions. The study sponsor and a subject advisory board reviewed and revised the survey for understandability and readability before cognitive pre-test interviews. The cognitive pre-test interviews were conducted individually with 66 subjects who met the eligibility criteria via telephone call and a secure desktop sharing platform. The cognitive pre-test interviews aimed to examine the understanding of the survey by eligible subjects; ensuring interpretation of survey questions was consistent, concepts were not ambiguous, and that the survey was viewed as manageable by subjects to minimize burden or error from fatigue. Revisions were made to the survey based on feedback received from subjects during the cognitive pre-test interviews prior to administering the survey. Phase 2 included administering the cross-sectional online survey, the results of which are reported herein. The full survey is available in the Supplementary materials.

Eligible subjects were invited to complete the survey with a nine-week data collection period. Subjects were recruited from multiple sources including patient panels, databases, patient associations, and physician referrals. The survey required approximately 20 min to complete and all questions were close ended. Subjects may have seen different questions based on their prior experience with rimegepant. The overall study sample included subjects with varying patterns of rimegepant use, including:

- STP use (STP-only use, STP and LTP use, STP and acute use, and STP, LTP, and acute use)
- LTP use (LTP-only use and LTP and acute use)
- Acute-only use

To maximize the STP cohort, a hierarchy was applied so that a prespecified subgroup of subjects who reported using rimegepant for STP treatment was found (Figure 1). The definition of STP use was derived from an expert consensus, where experts agreed on a treatment category referred to as “situational prevention” or “short-term prevention (19).” This was defined as treatment to prevent migraine attacks by taking medication before a reliable and predictable migraine trigger or for a time-limited period where headache avoidance is important. Other terms for STP include mini-prophylaxis and pre-emptive treatment. Acute use was defined as treatment to “stop, relieve, or lower symptoms when a migraine attack occurs.” LTP use was defined as treatment “to prevent or reduce the severity or frequency of migraine attacks by taking consistently/regularly (every other day) without breaks.”

**Figure 1:**
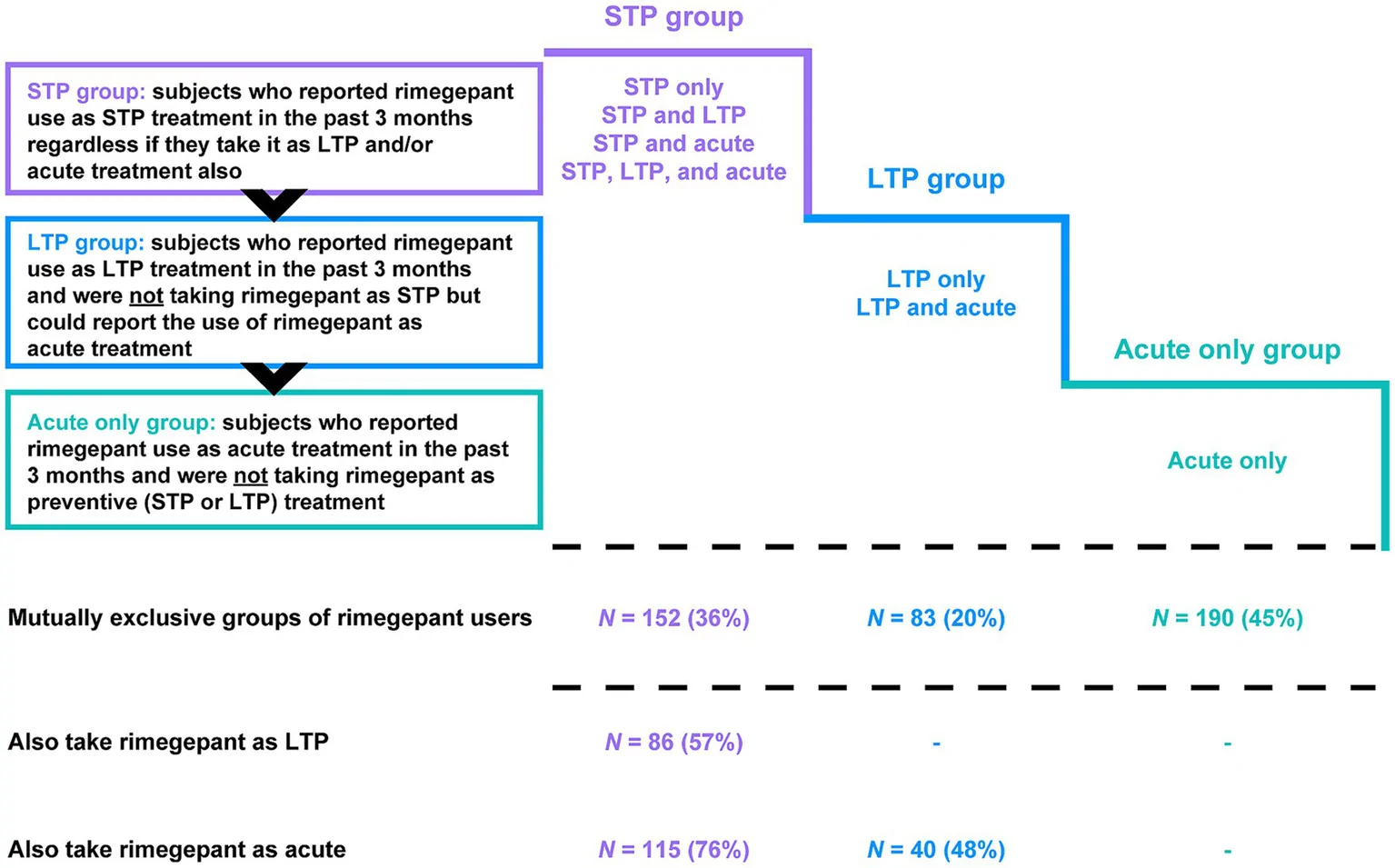
Sample hierarchy and mutually exclusive groups of rimegepant users.

### 2.2 Subjects

Eligible subjects were US residents aged 18 years or older who self-reported a diagnosis of episodic or chronic migraine and were currently receiving rimegepant treatment. Subjects also had initiated rimegepant at least 3 months prior to survey completion and had taken at least 1 dose of rimegepant within 30 days before completing the survey. The survey was administered in English only, and subjects self-reported their ability to read and understand English. Subjects were excluded if they were using more than 1 prescription preventive migraine treatment at the time of the survey, had participated in a clinical trial involving rimegepant, or were currently taking another gepant in addition to rimegepant. This study aimed to include a minimum number of males (≥75) and a minimum number of STP users (≥100). The terms “woman/female,” “man/male,” and ‘sex’ used herein refer to the biological sex of the individual.

### 2.3 Ethics statement

The final protocol, informed consent documentation, and study questionnaire were submitted to Pearl IRB (Indianapolis, IN), and an exemption determination was granted (according to 45 CFR 46.104(d) (2) Tests, Surveys, Interviews) on July 3, 2024 (IRB ID #: 2024–0281). All subjects were required to provide informed consent, and no personally identifiable information was captured. This study complied with all local regulatory and legal requirements, including applicable privacy laws.

### 2.4 Statistical analyses

This analysis was primarily exploratory and hypothesis-generating in nature. Subject demographics, health and migraine characteristics, comorbidities, treatment, treatment satisfaction and experience with rimegepant (STP) were reported descriptively, using mean and standard deviation (SD) for continuous variables and frequency (%) for categorical variables, respectively. Comparisons of categorical variables (survey responses) were compared by sex using Chi-square tests, with Fisher’s exact tests in cases where expected cell counts <5. A *p* value of 0.05 (2-tailed) was considered statistically significant for all comparisons. Reported *p* values are nominal (unadjusted).

## 3 Results

### 3.1 Subjects

Of 25,077 screened subjects, 425 adults with migraine were enrolled and no subjects discontinued. The most common reasons for screening failure were “never told by a healthcare provider that they have migraine” (*n* = 12,903; 52.4%), “not currently taking any migraine medication” (*n* = 4,971; 20.2%), and ‘current preventive migraine medication is not rimegepant’ (*n* = 4,234; 17.2%). Subject disposition is summarized in Supplemental Figure 1. Three mutually exclusive groups of rimegepant users were identified: STP users (*n* = 152); LTP-only users (*n* = 83), and acute-only users (*n* = 190; Figure 1). Of STP users, 15.8% (*n* = 24) only used rimegepant for STP. Rimegepant was usually prescribed during an in-person appointment (88%), followed by telehealth/phone or video chat (7%).

Subject demographics and clinical characteristics are summarized in Table 1. In the STP group, subjects had a mean (SD) age of 44.2 (10.2) years, 98 (64.5%) were female, 126 (82.9%) were White and the mean (SD) number of migraine days in the 30 days prior to the survey was 6.4 (5.6). The most common comorbidities among the STP group were anxiety (*n* = 91, 59.9%), depression (*n* = 69, 45.4%), and cluster headache (*n* = 55, 36.3%; Table 2). The prevalence of comorbidities in the STP group was generally similar to the total sample (Table 2).

**Table 1:** Subject demographics, disease history, and clinical characteristics.

Characteristic	STP (*N* = 152)	Total (*N* = 425)
Age, y	44.2 (10.2)	44.7 (11.1)
Sex, *n* (%)		
Male	54 (35.5)	108 (25.4)
Female	98 (64.5)	316 (74.4)
Height, in	66.1 (3.8)	65.9 (3.7)
Weight, pounds	177.4 (47.2)	180.2 (51.8)
BMI, kg/m^2^	28.5 (6.9)	29.2 (8.1)
Race, *n* (%)		
African American/Black	18 (11.8)	55 (12.9)
Asian	2 (1.3)	4 (0.9)
American Indian/Alaska Native	1 (0.7)	9 (2.1)
Native Hawaiian or other Pacific Islander	1 (0.7)	2 (0.5)
White	126 (82.9)	355 (83.5)
Other	8 (5.3)	18 (4.2)
Prefer not to say	0	1 (0.2)
Ethnicity, *n* (%)		
Hispanic or Latino	30 (19.7)	62 (14.6)
Not Hispanic or Latino	122 (80.3)	362 (85.2)
Prefer not to say	0	1 (0.2)
Time since migraine diagnosis, years	11.2 (12.8)	13.4 (13.2)
Number of headache days in the past 30 days, d	8.8 (6.9)	10.3 (7.1)
Number of migraine days in the past 30 days, d	6.4 (5.6)	6.9 (5.6)
Moderate or severe pain intensity,* *n* (%)	126 (82.9)	362 (85.2)
Nausea,* *n* (%)	99 (65.1)	260 (61.2)
Photophobia,* *n* (%)	125 (82.2)	351 (82.6)
Phonophobia,* *n* (%)	129 (84.9)	351 (82.6)

SD, standard deviation.

Data are mean (SD) unless stated otherwise. *Half the time or more.

**Table 2:** Comorbidities ever diagnosed by a healthcare provider and occurring in ≥5% in subjects receiving rimegepant for STP or the total sample.

Comorbidity	STP (*N* = 152)	Total (*N* = 425)
*n* (%)		
Anxiety	91 (59.9)	256 (60.2)
Depression	69 (45.4)	212 (49.9)
Cluster headache	55 (36.2)	135 (31.8)
Allergies/hay fever	40 (26.3)	143 (33.6)
Insomnia	39 (25.7)	119 (28.0)
Sleep apnea	37 (24.3)	107 (25.2)
High cholesterol	35 (23.0)	115 (27.1)
Hypertension	34 (22.4)	94 (22.1)
Asthma	29 (19.1)	90 (21.2)
Vitamin D deficiency	26 (17.1)	94 (22.1)
Arthritis, type unknown	25 (16.4)	78 (18.4)
Concussion	25 (16.4)	83 (19.5)
Diabetes	20 (13.2)	53 (12.5)
Thyroid disease	16 (10.5)	57 (13.4)
Rheumatoid arthritis	15 (9.9)	40 (9.4)
Osteoarthritis	13 (8.6)	56 (13.2)
Osteoporosis	9 (5.9)	33 (7.8)
Epilepsy	8 (5.3)	21 (4.9)
Psoriasis	8 (5.3)	27 (6.4)
Gastric ulcer/gastrointestinal bleeding	5 (3.3)	23 (5.4)

### 3.2 Migraine preventive treatment

The number of subjects who had previously taken prescription migraine preventive treatment was 65 (42.8%) in the STP group and 252 (59.3%) in the total sample, respectively. The mean (SD) time since first STP use of rimegepant was 1.8 (1.2) years, and the mean number of times taking rimegepant as STP in the last 30 days was 7.6 (5.5).

Current and previous preventive migraine treatments are summarized in Table 3. In the STP group, the most common previous prescription migraine prevention treatment was topiramate (*n* = 26, 40.0%). The most common type of preventive migraine treatment currently used in the STP group was over-the-counter treatments (*n* = 73, 48.0%). Most subjects learned about rimegepant as STP treatment through their prescribing physicians (*n* = 134, 88.2%; male = 45, 83.3%; female = 89, 90.8%). Overall, primary care providers (49%), general neurologists (25%), and headache specialists (15%) were the main rimegepant prescribers.

**Table 3:** Current and historical preventive migraine treatment history.

Migraine preventive treatment type,* *n* (%)	STP		Total	
	Current (*N* = 152)	Previous (*N* = 65)	Current (*N* = 425)	Previous (*N* = 252)
Over-the-counter treatment	73 (48.0)	—	171 (40.2)	—
Prescription oral treatment	36 (23.7)	—	132 (35.9)	—
Home remedies (e.g., massages)	44 (28.9)	—	138 (32.5)	—
Injections from my healthcare provider (e.g., Botox)	8 (5.3)	—	42 (11.4)	—
Prescription self-injection treatment	5 (3.3)	—	35 (9.5)	—
Behavioral treatments	10 (6.6)	—	31 (7.3)	—
A device to prevent or reduce migraine attacks (e.g., an implant or external stimulator device)	10 (6.6)	—	13 (3.1)	—
Infusion	0	—	2 (0.5)	—
Surgery to prevent or reduce migraine attacks	1 (0.7)	—	2 (0.5)	—
I do not use any products besides rimegepant to prevent migraine attacks	38 (25.0)	—	94 (22.1)	—
Migraine preventive treatment prescription, *n* (%)				
Rimegepant, for prevention	152 (100)	9 (13.8)	235 (55.3)	54 (21.4)
Topiramate	0	26 (40.0)	38 (8.9)	119 (47.2)
OnabotulinumtoxinA	0	11 (16.9)	23 (5.4)	38 (15.1)
Fremanezumab	0	4 (6.2)	19 (4.5)	18 (7.1)
Erenumab	0	3 (4.6)	8 (1.9)	30 (11.9)
Amitriptyline	0	8 (12.3)	8 (1.9)	32 (12.7)
Galcanezumab	0	4 (6.2)	8 (1.9)	25 (9.9)
Eptinezumab	0	2 (3.1)	1 (0.2)	4 (1.6)
Atogepant	0	3 (4.6)	0	10 (4.0)
Another prescription treatment	0	14 (21.5)	28 (6.6)	48 (19.0)
I do not currently use any prescription preventive treatment	0	3 (4.6)	57 (13.4)	8 (3.2)

*Answers in response to “In addition to rimegepant, use or plan to use any of the following to prevent migraine attacks from occurring”.

### 3.3 Why subjects took rimegepant as STP treatment

Overall, the most common reason subjects took rimegepant as an STP treatment was because it was recommended by their doctor (*n* = 94, 61.8%; Figure 2). Compared with male subjects, female subjects were more likely to use rimegepant as STP treatment due to doctor recommendation (48.1% vs. 69.4%, *p* = 0.014), frequency of migraine attacks (25.9% vs. 43.9%, *p* = 0.036), and severity of migraine attacks (22.2% vs. 43.9%, *p* = 0.008; Figure 2).

**Figure 2:**
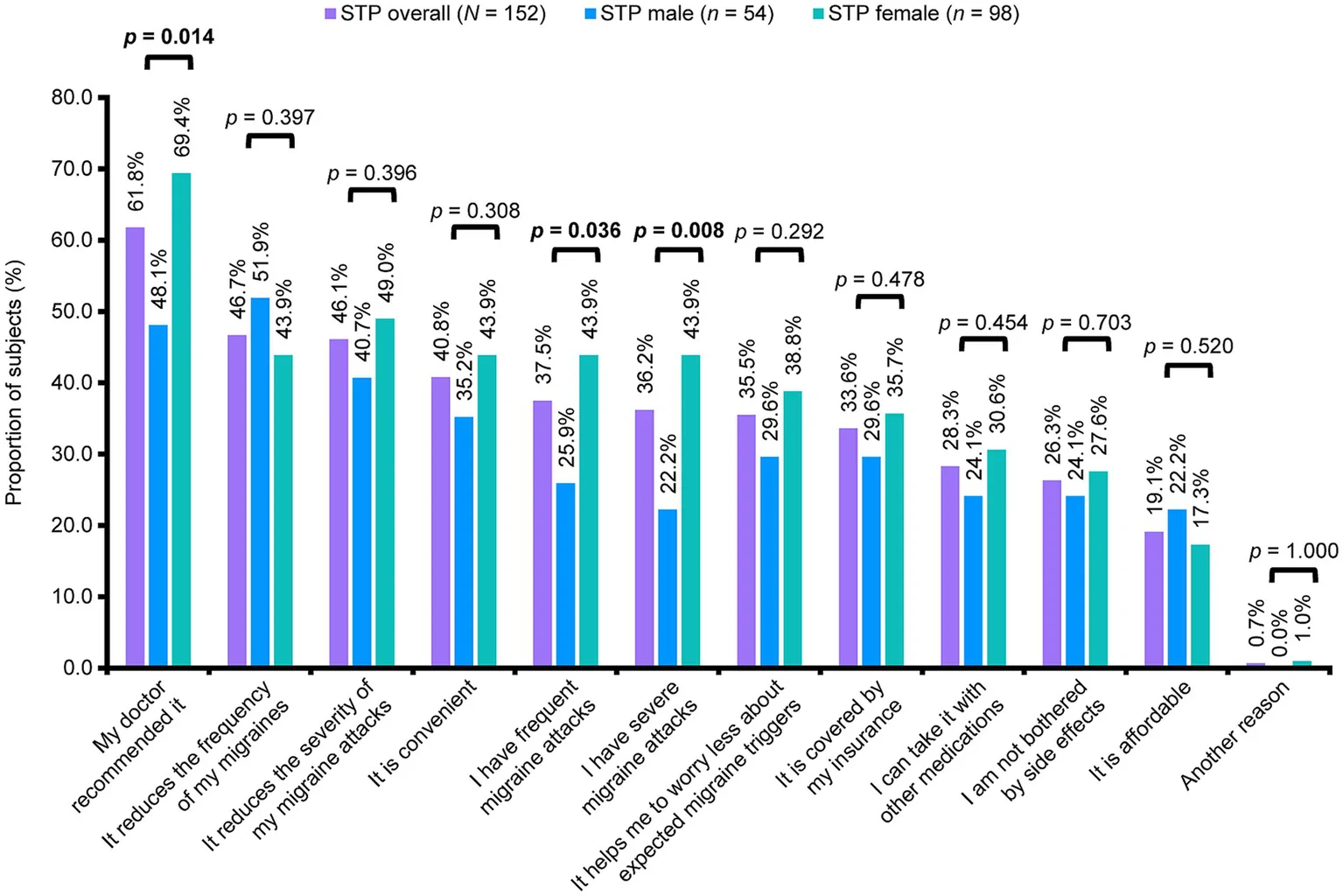
Reasons identified by male and female subjects for taking rimegepant as a STP treatment for migraine. Answers in response to “Why do you take rimegepant as a short-term prevention treatment?”.

Overall, 64.5% (*n* = 98) of subjects took rimegepant as STP treatment to prevent additional migraine attacks after experiencing a migraine (Figure 3). There was no significant difference between male and female subjects for the situations in which they took rimegepant as STP treatment (Figure 3).

**Figure 3:**
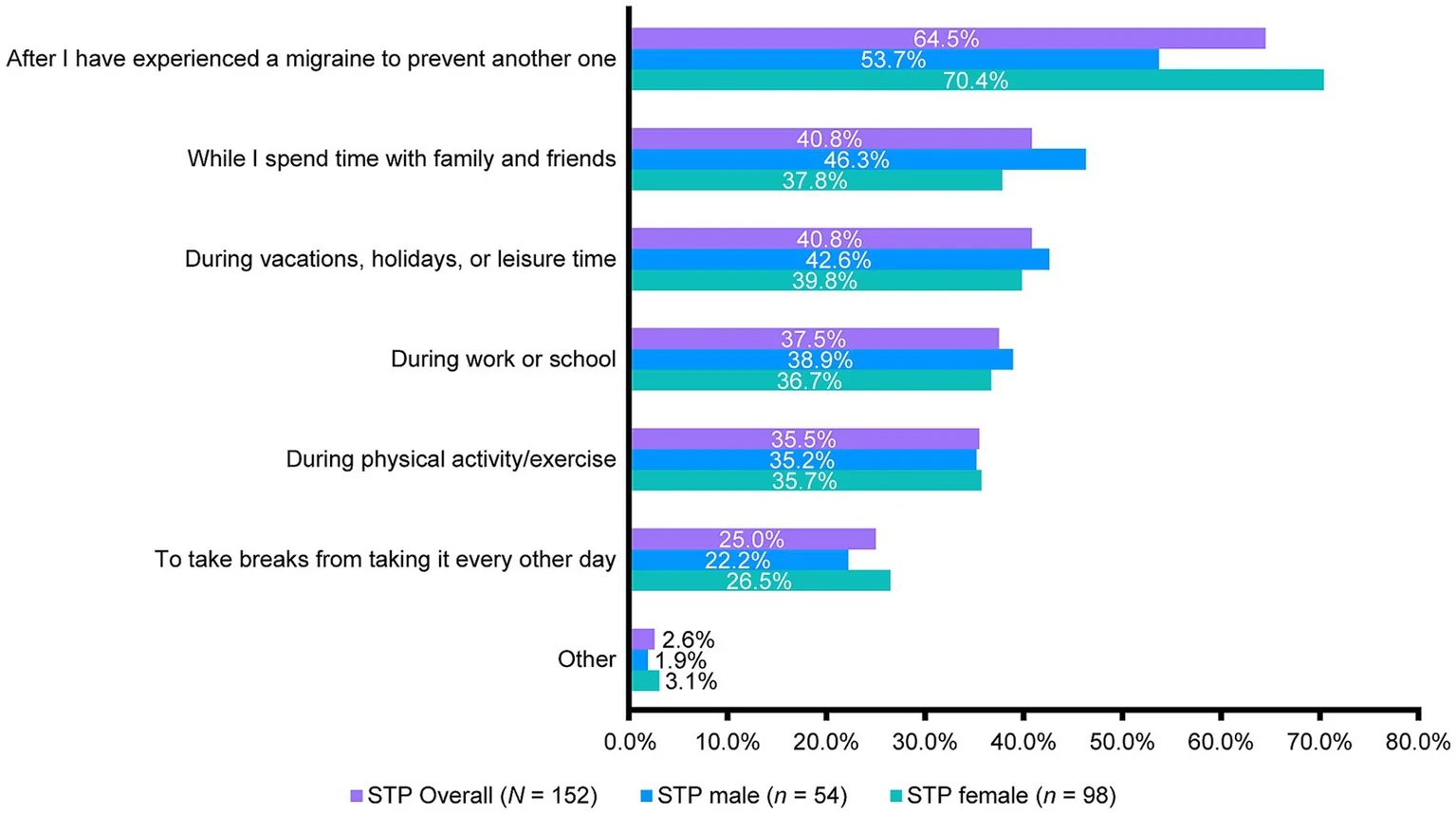
Situations during which rimegepant is taken as STP treatment to reduce the likelihood of migraine attacks. Answers in response to “During which of the following situations do you sometimes take rimegepant as a short-term prevention treatment to reduce the likelihood of migraine attacks?”.

Overall, stress (*n* = 105, 69.1%) and disruptive sleeping patterns (*n* = 71, 46.7%) were the most common factors identified as provocative of a migraine attack (Figure 4). In female subjects, period/menstrual cycle (30.9%) was one of the most common factors reported. Additionally, among female subjects, hormonal changes (29.6% vs. 7.4%, *p* = 0.002), disruptive sleep pattern (53.1% vs. 35.2%, *p* = 0.042), light (48.0% vs. 27.8%, *p* = 0.017), and dehydration (39.8% vs. 22.2%, *p* = 0.032) were more common than in male subjects (Figure 4). In male subjects, sexual activity as a factor provocative of a migraine attack was more common than in female subjects (14.8% vs. 3.1%, *p* = 0.017; Figure 4).

**Figure 4:**
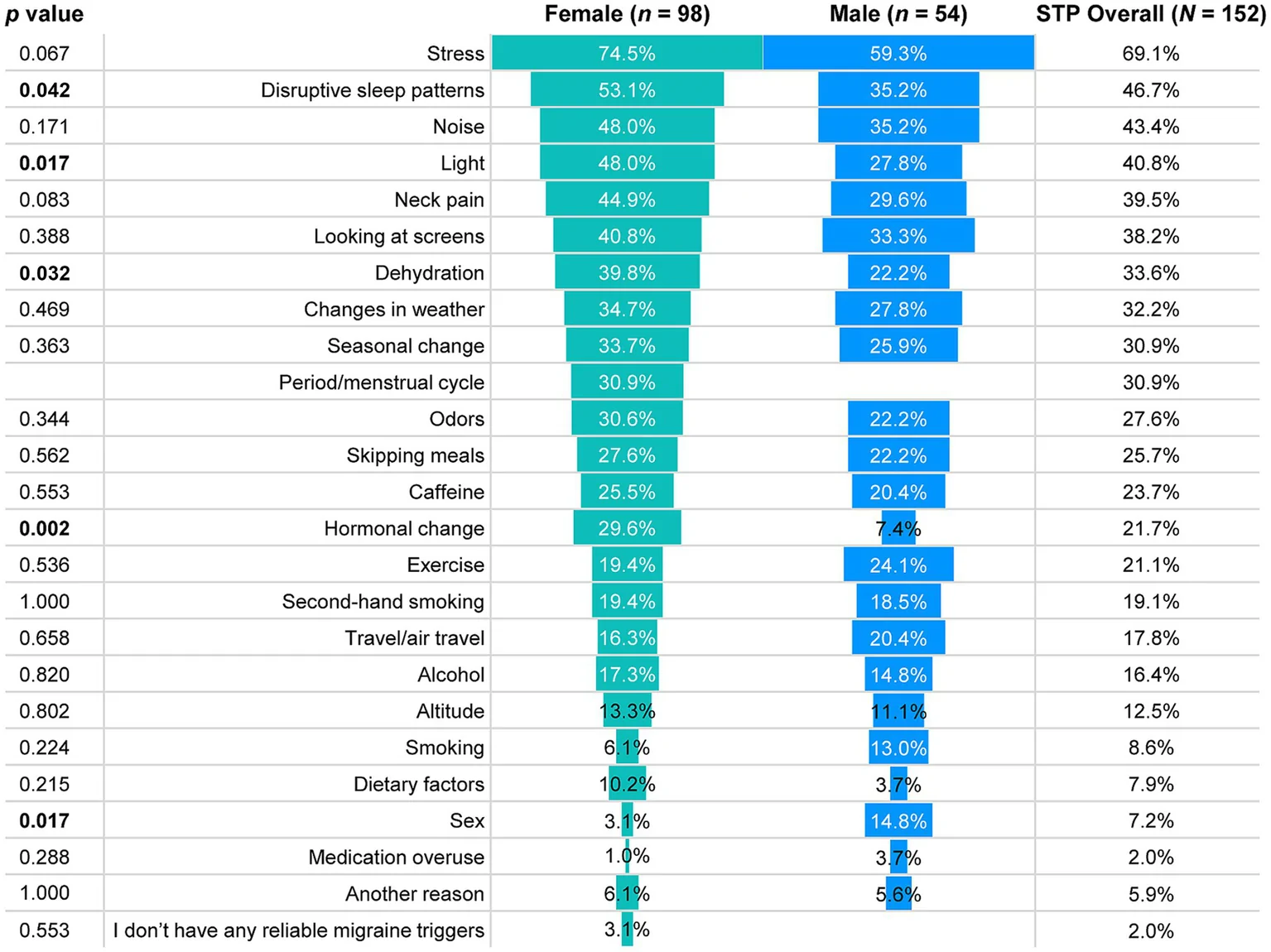
Factors provocative of a migraine attack in male and female subjects for which rimegepant was taken as a STP preventive treatment. Answers in response to “Which of the following, if any, are migraine triggers for which you have taken or plan to take rimegepant as a short-term prevention treatment?”.

### 3.4 How rimegepant was taken as STP treatment

Overall, 36.2% (*n* = 55), 37.5% (*n* = 57), and 42.1% (*n* = 64) took rimegepant as STP treatment one time, every day, and every other day, respectively (Figure 5A). There was no difference among male and female subjects in the frequency of rimegepant when taken when used as STP treatment (Figure 5A). When using rimegepant as STP treatment, approximately half of subjects took it for 2−3 days, regardless of biological sex (Figure 5B).

**Figure 5:**
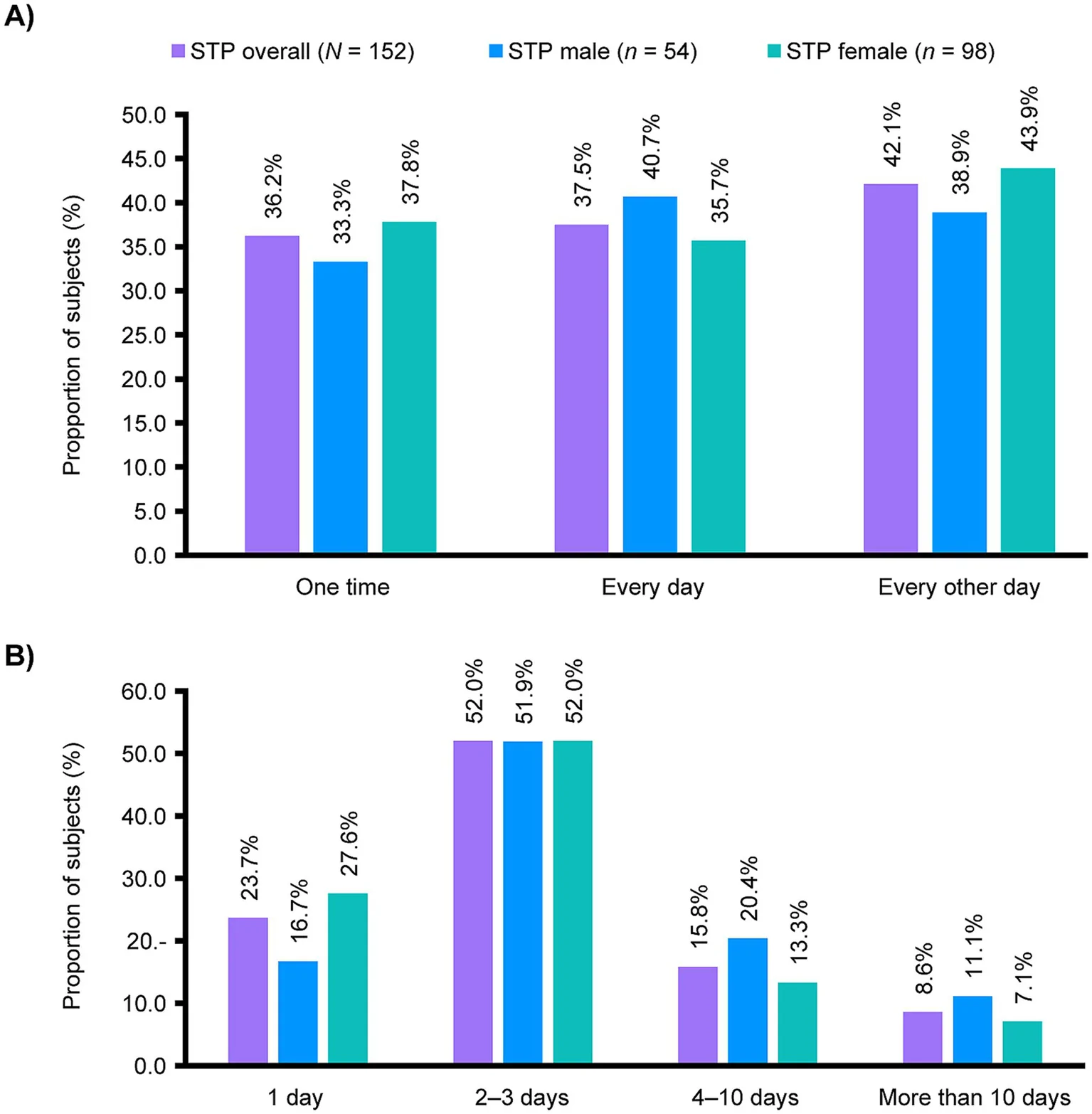
Frequency **(A)** and duration **(B)** of when rimegepant is taken as a STP treatment for migraine in male and female subjects. Answers in response to: **(A)** “When you have taken rimegepant as a short-term prevention treatment, how have you taken it?” **(B)** “Thinking about the last time you took rimegepant as a short-term prevention treatment, for how long did you take it before you stopped?” For **(A)**, subjects could have selected >1 answer, for **(B)** only subjects could only select 1 response.

### 3.5 Satisfaction with rimegepant as an STP treatment

Overall, most subjects were “satisfied” to “extremely satisfied” in using rimegepant as an STP treatment for one time among those who responded (80.0% [44/55], Figure 6A), everyday (77.2% [44/57], Figure 6B), and every other day (76.6% [49/64]; Figure 6C). Male subjects reported numerically higher levels of satisfaction (“satisfied - extremely satisfied” vs. other response) with taking rimegepant as an STP treatment one time than female subjects (94.4% [17/18] vs. 73.0% [27/37], *p* = 0.08). However, this difference was not statistically significant.

**Figure 6:**
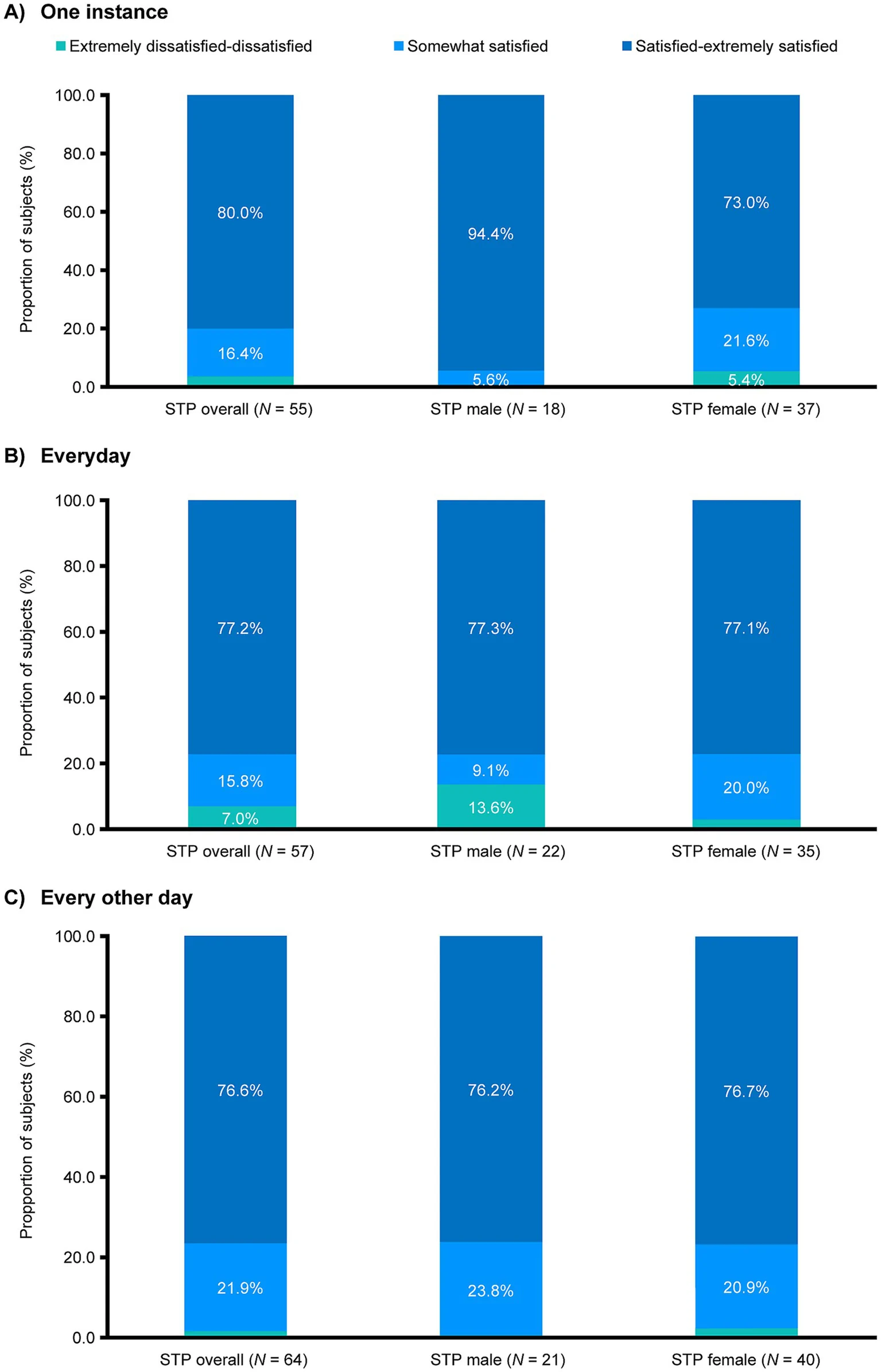
Satisfaction in male and female subjects with posology taking rimegepant as a STP treatment at a frequency of **(A)** one instance **(B)** everyday and **(C)** every other day. Answers in response to “How satisfied or dissatisfied are you with taking rimegepant as a short-term prevention treatment?” Proportions <5% are not labeled.

## 4 Discussion

This cross-sectional observational study is the first to provide real-world evidence of utilization of rimegepant as STP treatment for migraine. Although objective clinical endpoints, such as changes in monthly migraine days were not captured, subjects expressed high levels of satisfaction with rimegepant as STP treatment, whether taken one time, every day, or every other day. As the frequency and severity of migraine attacks can fluctuate over time, as well as in response to specific identifiable factors or events, treatment needs will also vary over time. There may also be periods of time where a desire for freedom from migraine is independent of attack frequency or severity. In this respect, STP treatment may help fill unmet needs for greater flexibility in migraine treatment, especially among those who do not wish to take continuous LTP treatment. One example of where the frequency and severity of migraine attacks can fluctuate over time is menstrual-related migraine, which is characterized by increased migraine frequency and severity during the perimenstrual window. In a case series of 5 women with episodic migraine and regular menstrual cycles, rimegepant 75 mg given every other day as STP treatment (initiated 2 days prior to menstruation and continued until 2 days post menstruation) prevented perimenstrual attacks in 17/20 cycles (85%) with no treatment-related adverse events (24). Similar to our study, this case series reported high levels of satisfaction with rimegepant as STP treatment. Traditional STP treatment options, including non-steroidal anti-inflammatory drugs or triptans, often show insufficient response and may increase the risk of gastrointestinal complications, medication overuse, or potentially vascular adverse events (29). As such, there is a potential unmet medical need for effective STP treatment with less risk of adverse events that are associated with traditional STP treatments. Our study provides support for further investigation with rimegepant as STP treatment.

Offering STP treatment intrinsically implies a higher degree of patient participation in formulating an individualized treatment plan, which may lead to other benefits, such as a greater patient participation in wellness programs and, potentially, improved migraine control (30). This study further reinforces the importance of the doctor-patient relationship, with almost 90% of subjects learning about rimegepant as STP treatment from their prescribing physician and nearly two thirds of subjects taking rimegepant as STP treatment because it was recommended by their doctor. In this study, over a third of subjects were using rimegepant as STP only, although additional acute and preventive use was also common. Effective communication between clinicians and patients is integral to optimal medical care, including providing accurate diagnoses and creating effective treatment plans; however, in a survey of US-based neurologists, dialogue with many of their subjects with migraine did not assess elements essential for diagnosis and treatment (31). This has important implications for effective migraine management, as well as progression. In a 1-year time series analysis, a cyclic migraine phenotype was associated with migraine progression after 1 year (32). This was particularly evident in subjects with episodic migraine and a cyclic phenotype and inadequate preventive therapy, with their headache frequency increasing over time (32). Preventive treatment appeared to play a fundamental role in modulating cyclic migraine occurrence. As such, STP treatment at times of attack clusters warrants further investigation.

Migraine remains a predominantly female disorder with a clear link to the reproductive lifespan, including a sharp rise in incidence after onset of menarche and a decline after menopause. Data from the Migraine in America Symptoms and Treatment (MAST) Study, a US longitudinal study, reported that compared with men, women were younger, had more monthly headache days, and were more likely to report moderate or severe headache-related disability and cutaneous allodynia (6). Furthermore, pain features (phonophobia, photophobia, nausea) and the lifetime rate of medical consultation for headache were significantly higher in women than in men (6). In our study, several sex differences in rimegepant STP uses were evident. Female subjects were significantly more likely to take rimegepant as STP treatment due to doctor recommendation, as well as in response to more frequent and severe migraine attacks. In female subjects, period/menstrual cycle was the most common factor to cause a migraine attack, and disruptive sleep pattern, light, and dehydration were more common than in male subjects, whereas sexual activity as a factor provocative of a migraine attack was more common in male than in female subjects. Our results were generally consistent with previous research. In a cross-sectional study of over 6,000 subjects with migraine, bright light, stress, skipping a meal, sleep deprivation, high altitudes, and weather changes were more often reported as factors precipitating a migraine attack in women than in men (18). Furthermore, women report more factors provocative of a migraine attack than men. The higher rate of factors precipitating a migraine attack in female vs. male subjects is likely to partly stem from the role of female sex hormones in the pathophysiological mechanism of migraine. Although other hormones exert an effect, the influence of estrogen on susceptibility to a migraine attack is well documented. (33). Women may be predisposed to a migraine attack during times of unstable and rapidly declining estrogen levels, whereas high and stable levels of estrogen can, under certain conditions, exert a protective effect (11). With relevance to rimegepant and other CGRP-targeting preventive treatments, higher plasma CGRP levels are observed in women compared with men, indicating that CGRP and estrogen systems may be intertwined (10). Given the importance of these factors, it is crucial to understand the relationship between migraine management the role of female sex hormones, menstrual cycles, and perimenopause in triggering disabling and treatment-resistant migraine attacks (33). Notably, migraine severity and frequency often worsen during the menopausal transition due to fluctuating estrogen levels. In contrast, stabilization of estrogen and other hormones after menopause results in an improvement in migraine for many (but not all) women (33). Changes in sex hormones are unlikely to be the only contributing factor because female subjects with migraine report more factors provocative of a migraine attack than male subjects, even when menstruation is disregarded (18). It is unclear why sexual activity as a factor provocative of a migraine attack was more common in male than in female subjects. Similarly, the pathophysiology of primary headache associated with sexual activity, a distinct diagnosis from migraine, is still debated (34). Primary headache associated with sexual activity is also more common in men than in women and may be accompanied by migraine, hypnic headache, or hemicrania continua, which may suggest a common mechanism (34).

Despite differences between male and female subjects in factors provocative of a migraine attack, there were no differences in the frequency or duration for which rimegepant was taken as STP treatment. With the exception of doctor recommendation and migraine frequency and severity, males and females took rimegepant for similar reasons and reported high levels of satisfaction. However, it should be noted that female subjects were not specifically asked if they took rimegepant as STP for menstrual migraine. As period/menstrual cycle was the most common factor likely to cause a migraine attack in female subjects, it is likely that some female subjects were taking rimegepant for menstrual migraine. In this case, female subjects who may have been taking rimegepant as STP for menstrual migraine are likely to have chosen the closest matching response, such as “recommended by their doctor” or “I have frequent migraine attacks.” This may partly account for some of the differences seen between why male and female subjects took rimegepant as STP treatment. Preventive anti-CGRP treatments, including fremanezumab, erenumab, eptinezumab, galcanezumab, and atogepant have previously showed comparable efficacy in men and women, despite low male representation in clinical trials (12–17). However, pooled analyses of acute treatments suggest that sex differences may exist for gepants, such as ubrogepant and rimegepant, with more favorable outcomes observed in females than in males for the endpoints 2-h pain freedom and 2-h freedom from most bothersome symptom (17). These differences may be the result of sex differences in the role of CGRP in maintaining an ongoing headache.

These findings should be interpreted in the context of the study limitations. The statistical analysis approach was primarily exploratory and hypothesis-generating rather than confirmatory, and relatively small sample sizes prevented the inclusion of robust multivariable or sensitivity analyses. Notably, only a small proportion of subjects used rimegepant for STP-only (15.8%). Results therefore largely represent STP as part of a more comprehensive treatment approach, including acute and/or LTP treatment, and limits attribution of certain outcomes, such as satisfaction, to STP alone. This study establishes neither rimegepant efficacy as STP treatment nor supports off-label use. Data were self-reported and could not be independently verified, therefore there is potential for recall bias or misreporting. The impact of rimegepant treatment on commonly used efficacy measures, such as changes in weekly or monthly migraine days, was not captured, and efficacy cannot be inferred from outcomes, such as subject satisfaction. Generalizability of the results may be limited, as STP users could have also been using rimegepant for acute and/or LTP treatment; however, not all STP users took rimegepant as acute treatment. As with many real-world studies, generalizability may also be influenced by healthcare-seeking behavior and follow-up patterns. Further research is warranted to evaluate the efficacy of STP and its potential impact on quality of life. We observed an approximately 60% comorbidity rate of anxiety in the STP cohort, which is relatively high in the context of published migraine studies. Anxiety is a well-established migraine comorbidity, which appears to have a bidirectional relationship with migraine, where migraine incidence increases the risk of anxiety and vice versa (35). Anxiety is also associated with exacerbated migraine progression, medication overuse, stigma, and migraine-related disability (35). Anxiety can worsen the fear of pain, which is common in individuals who experience recurrent headache; this may, in turn, lead to an intensification of behaviors to avoid headache/migraine triggers, which can have the converse effect of worsening migraine disability and progression (35–37). It is possible that anxiety or fear of pain contributed to the use of rimegepant as STP in our study. Future analysis adjusting for baseline anxiety as a confounder may help clarify whether satisfaction with STP is influenced by anxiety and to what extent anxiety drives patient choice in opting for STP. It should be noted that subjects in the STP group also reported a relatively high comorbidity of cluster headache (55/152; 36.2%). Migraine and cluster headache are distinct primary headache types, yet share numerous pathological features, pharmacological responses, as well as a potentially common pathophysiological pathway in the trigeminovascular system (38). In this instance it is possible that the term “cluster headache” may have been misunderstood. Subjects would have selected “cluster headache” from a list of health conditions in response to the question “Has a healthcare provider ever told you that you have any of the following health conditions?” It’s therefore not clear if subjects had true cluster headaches or clustering of migraine attacks across a number of days, which may have prompted use of rimegepant. Potential diagnostic misclassification should be considered in the interpretation of these results. The terms “male” and “female” used in this study carry inherent limitations, as is common to the majority of migraine studies, as they do not distinguish between sex assigned at birth and gender identity. As such, certain hormonal and psychological factors that may be relevant are not taken into account. In addition, little is known with regards to sex differences and treatment outcomes in specific patient populations including those who are transgender, elderly, those with migraine-related complications, children, and those receiving hormonal treatments. Finally, generating further data on the efficacy of rimegepant and other CGRP-targeting therapies in men may broaden the clinical value of these therapies and improve treatment management.

## 5 Conclusion

Female subjects were more likely than male subjects to use rimegepant as STP treatment due to doctor recommendation, as well as for frequency and severity of migraine attacks. Despite differences in factors provocative of migraine attacks, male and female subjects reported high levels of satisfaction with rimegepant as STP treatment. Long-term prospective studies are needed to evaluate the efficacy of STP, its impact on quality of life, hormonal impact, and the potential to prevent migraine progression.

## Data Availability

Upon request, and subject to review, Pfizer will provide the data that support the findings of this study. Subject to certain criteria, conditions and exceptions, Pfizer may also provide access to the related individual de-identified participant data. See https://www.pfizer.com/science/clinical-trials/trial-data-and-results for more information.

## Footnotes

1
https://sago.com/en/

2
oracle.com/life-sciences/
