# Supplementary material for: Sex differences in the real-world use of rimegepant for short-term prevention of migraine in the United States
Source: Front Neurol. 2026 Sep 24;17:1901123. doi: 10.3389/fneur.2026.1901123 (PMC13645540; doi:10.3389/fneur.2026.1901123)
Supplement: Supplementary file 1 [file Supplementary_File_1.DOCX]

**Supplemental materials**

**SUPPLEMENTAL FIGURE 1.** Subject disposition.

^a^Subjects may fail to meet ≥1 eligibility criteria.

^b^Subjects removed after data cleaning process due to data inconsistencies.
