# Supplementary material for: Sex differences in the real-world use of rimegepant for short-term prevention of migraine in the United States
Source: Front Neurol. 2026 Sep 24;17:1901123. doi: 10.3389/fneur.2026.1901123 (PMC13645540; doi:10.3389/fneur.2026.1901123)
Supplement: Supplementary file 2 [file Supplementary_File_2.DOCX]

**Rimegepant Patient Study**
**Patient Screener**

**Quantitative Phase**

| **Quota Groups** | **Quant n** |
| --- | --- |
| **Total Nurtec Patients** | **500** |
| Patients Using Nurtec as Short-term Prevention | 100 |
| Males | 75 |

**Objectives:**

1. Describe those using rimegepant for short-term prevention, long-term prevention, or acute treatment of migraine in terms of demographics, clinical characteristics and co-morbidities, disease history, and migraine treatment history
2. Assess treatment satisfaction among those using rimegepant
3. Assess how current rimegepant patients are using their treatment, in terms of frequency, duration, timing in relation to migraine symptoms and triggers, and reasons for taking rimegepant
4. Compare treatment use, treatment history, disease history, health characteristics, and demographics among short-term prevention, long-term prevention, and acute rimegepant users
5. Among those not currently using rimegepant for short-term prevention treatment:
a) Assess likelihood of using rimegepant as short-term prevention treatment
b) Assess treatment-related needs that may be addressed by using rimegepant as short-term prevention treatment
c) Describe the specific situations in which they envision using it, as well as potential barriers to using rimegepant as short-term prevention treatment

**Inclusion Criteria**

1. 18 years of age and older
2. Diagnosed with migraine (self-reported)
3. Currently taking rimegepant for their migraine
4. Started taking rimegepant at least 3 months prior to the administering of the survey
5. Have taken rimegepant within the past 30 days prior to the administering of the survey
6. Resides in the US
7. Self-reported ability to read and understand English
8. Evidence of a personally signed (or acknowledged if obtained electronically or online) and dated informed consent document indicating that the respondent has been informed of all pertinent aspects of the study.

**Exclusion criteria**

1. Unwilling or unable to provide informed consent
2. Currently take another gepant in addition to rimegepant
3. Participated in rimegepant clinical trials
4. Are using more than one preventive medication
5. For the cognitive interviews only, patients will be excluded if they indicate or exhibit speaking or hearing difficulties, which would make a telephone conversation challenging.

**SCREENER**

**SHOW EVERYONE:**

Thank you for your interest in this study. We will start by asking you a few questions.

**ASK EVERYONE**

S1a.	What is your age?

**ENTER WHOLE NUMBER;**

______ years

**TERMINATE IF <18**

S1b.	What is your gender?

| **SELECT ONLY ONE** | |
| --- | --- |
| 1 | Male |
| 2 | Female |
| 3 | Non-binary |
| 4 | A gender not listed |
| 98 | Prefer not to answer |

S2.	Has a healthcare provider **ever** told you that you have any of the following health conditions?

| **SELECT ALL THAT APPLY** | | |
| --- | --- | --- |
| 1 | Cluster headache |  |
| 2 | Concussion |  |
| 3 | Lupus |  |
| 4 | Migraine | **MUST SELECT** |
| 5 | Multiple sclerosis |  |
| 6 | Osteoporosis |  |
| 7 | Sleep apnea |  |
| 8 | Thyroid disease |  |
| 99 | No, none of the above **[EXCLUSIVE]** | **TERMINATE** |

**TERMINATE IF S2≠4 “MIGRAINE”**

**FLAG IF ≥5 ARE SELECTED**

S2b.	Please describe the pain and other symptoms you have with your headaches. If you have more than one type of headache, please answer for your most **severe** type of headache. How often do you experience each of the following symptoms that associated with your most severe type of headache?

| **SELECT ONE RESPONSE FOR EACH** | | **Never** | **Rarely** | **Less Than Half the Time** | **Half the Time or More** |
| --- | --- | --- | --- | --- | --- |
| a | The pain is worse on one side | 1 | 2 | 3 | 4 |
| b | The pain is pounding, pulsating or throbbing | 1 | 2 | 3 | 4 |
| c | The pain has moderate or severe intensity | 1 | 2 | 3 | 4 |
| d | The pain is made worse by routine activities such as walking or climbing stairs | 1 | 2 | 3 | 4 |
| e | You feel nauseated or sick to your stomach | 1 | 2 | 3 | 4 |
| f | Light bothers you (more than when you do not have headaches) | 1 | 2 | 3 | 4 |
| g | Sound bothers you (more than when you do not have headaches) | 1 | 2 | 3 | 4 |

**PROGRAMMER: PARTICIPANT MUST SELECT 2 OR MORE OF 3 ‘LESS THAN HALF THE TIME’ OR 4 ‘HALF THE TIME OR MORE’ TO ANY A-D OPTIONS**

**AND 3 ‘LESS THAN HALF THE TIME’ OR A 4 ‘HALF THE TIME OR MORE’ TO EITHER NAUSEA (E) OR BOTH PHOTOPHOBIA (F) AND PHONOPHOBIA (G). OTHERWISE TERMINATE**

S2a.	Do you **currently** take any **prescription** medication to stop, relieve, lower symptoms of, or prevent migraine attacks?

| **SELECT ONE** | | |
| --- | --- | --- |
| 1 | Yes |  |
| 2 | No | **TERMINATE** |

S3.	Some people take **medication** to stop, relieve or lower symptoms **when a migraine attack occurs**. This is also known as **acute** treatment for migraine.

Which, if any, of the following **prescription medications** do you **currently** use or have recently used (in the past month) for the **acute** treatment for migraine?

Please **do not** include any medications you take to prevent migraine; we will ask you about those next.

| **SELECT ALL THAT APPLY** | |
| --- | --- |
| 1 | Amerge^®^ (naratriptan) |
| 2 | FROVA^®^ (frovatriptan) |
| 3 | IMITREX^®^, Sumavel^®^ DosePro^®^, Tosymra^®^, Zembrace^®^, etc. (sumatriptan) |
| 4 | Nurtec^®^ ODT (rimegepant) – for acute |
| 5 | Relpax^®^ (eletriptan) |
| 6 | Reyvow^®^ (lasmiditan) |
| 7 | UBRELVY^®^ (ubrogepant) **[TERMINATE]** |
| 8 | ZAVZPRET™ (zavegepant) **[TERMINATE]** |
| 9 | ZOMIG^®^ (zolmitriptan) |
| 97 | Another prescription medication **[EXCLUSIVE]** |
| 99 | I don’t currently use or plan on using any **acute** prescription medications **[EXCLUSIVE]** |

S4.	Some people take **medications** consistently/regularly (not for a limited period of time) to prevent or reduce the severity or frequency of migraine attacks. This is also known as **preventive medication**.

Which, if any, of the following **prescription medications** do you **currently** use as **preventive medication?** Please **do not** include any medications you take exclusively for the acute treatment migraine.

| **SELECT ALL THAT APPLY** | |
| --- | --- |
| 1 | Aimovig^®^ (erenumab) |
| 2 | Ajovy^®^ (fremanezumab) |
| 3 | Botox^®^ (onabotulinumtoxinA) |
| 4 | Elavil^®^ (amitriptyline) |
| 5 | Emgality^®^ (galcanezumab) |
| 6 | Nurtec^®^ ODT (rimegepant) – for prevention |
| 7 | Qulipta^®^ (atogepant) **[TERMINATE]** |
| 8 | Topamax^®^ (topiramate) |
| 9 | Vyepti^®^ (eptinezumab) |
| 97 | Another prescription medication **[EXCLUSIVE]** |
| 99 | I don’t currently use any prescription **preventive medication [EXCLUSIVE]** |

**MUST SELECT “Nurtec ODT (Rimegepant)” TO CONTINUE (S3=4 OR S4=6)**

**CAN SELECT ONLY ONE PREVENTION TREATMENT OR 97, OTHERWISE TERMINATE**

**PROGRAMMER: SHOW S3 AND S4 ON THE SAME SCREEN**

S5.	When was the **first** time you took Nurtec^®^ ODT (rimegepant)?

Please make your best guess if you are unsure.

**SHOW DROP-DOWN FOR “MONTH” AND “YEAR”.**

**RANGE 2020-CURRENT YEAR**

**IF MONTH + YEAR IS LESS THAN 3 MONTHS FROM CURRENT MONTH, TERMINATE**

S5a.	Have you participated in any Nurtec^®^ ODT (rimegepant) clinical trials?

| **SELECT ONE** | | |
| --- | --- | --- |
| 1 | Yes | **TERMINATE** |
| 2 | No |  |

S6.	When was the **last** time you took Nurtec^®^ ODT (rimegepant)?

| **SELECT ONLY ONE** | |
| --- | --- |
| 1 | 30 days ago or less |
| 2 | 31-60 days ago |
| 3 | 61-90 days ago |
| 4 | More than 3 months ago |
| 99 | I don’t recall |

**IF S6=2-99, TERMINATE**

S6a.	How many days ago was the **last** time you took Nurtec^®^ ODT (rimegepant)?

Please make your best guess if you are unsure. If you took it today, enter “0”

| **SELECT ONLY ONE** | |
| --- | --- |
| 1 | ____ days ago **RANGE = 0-30** |

S7.	Some people use Nurtec^®^ ODT (rimegepant) in the following ways:

1. **ACUTE**: The goal is to stop, relieve, or lower symptoms when **a migraine attack occurs**.
2. **SHORT-TERM PREVENTION**: To prevent migraine attacks by taking medication for a limited period of time before a known **migraine trigger** **HOVER FOR MIGRAINE TRIGGER DEFINITION** or a specific situation when you want to reduce the chances of having a migraine attack.

**HOVER FOR DEFINITION:** Migraine Trigger: An external or internal factor (e.g., stress, poor sleep quality, diet, dehydration and/or alcohol/caffeine, etc.) that can cause a migraine attack either by itself or in combination with other factors.

3. **LONG-TERM PREVENTION:** To prevent or reduce the severity or frequency of migraine attacks by taking consistently/regularly (every other day) without breaks.

Please review the below figure for examples of what is meant by acute, short-term prevention and long-term prevention:

In the **past 3 months**, in which ways have you used Nurtec^®^ ODT (rimegepant)?

| **SELECT ALL THAT APPLY** | |
| --- | --- |
| 1 | Acute |
| 2 | Short-Term Prevention |
| 3 | Long-Term Prevention |

**ACUTE – S7 = 1**

**SHORT-TERM PREVENTION – S7 = 2**

**LONG-TERM PREVENTION – S7 = 3**

**RESPONDENTS CAN BE IN MORE THAN ONE GROUP.**

**ADD ERROR MESSAGE:**

- **S3 = 4 NURTEC AND S7 ≠ 1 ACUTE:**

“Earlier you indicated that you use Nurtec^®^ ODT (rimegepant) for acute treatment, please review your selections before continuing”

- **S3 ≠ 4 NURTEC AND S7 = 1 ACUTE:**

“Earlier you indicated that you **do not** use Nurtec^®^ ODT (rimegepant) for acute treatment, please review your selections before continuing”

- **S4 = 6 NURTEC AND S7 ≠ 2 SHORT-TERM PREVENTION OR 3 LONG-TERM PREVENTION:**

“Earlier you indicated that you use Nurtec^®^ ODT (rimegepant) for preventive treatment, please review your selections before continuing”

- **S4 ≠ 6 NURTEC AND S7 = 2 SHORT-TERM PREVENTION OR 3 LONG-TERM PREVENTION:**

“Earlier you indicated that you **do not** use Nurtec^®^ ODT (rimegepant) for preventive treatment, please review your selections before continuing”

**IF S7=2 SHORT-TERM PREVENTION SELECTED, ADD FLAG IN DATA FOR REPORTING**

**IF DISQUALIFIED, DISPLAY THE FOLLOWING**

Thank you so much for your time. Based on your responses, your profile does not match the survey eligibility criteria and we cannot invite you to participate at this time. However, we greatly appreciate your willingness to participate and hope that we can provide another opportunity for you to do so in the future.

**IF QUALIFIED, DISPLAY THE FOLLOWING**

Thanks, you have qualified for the study.

**END SCREENER**

**Rimegepant Patient Study**
**Patient Questionnaire**

**Quantitative Phase**

**Section A: Patient-Reported Outcomes**

Thank you for participating in this survey! The following questions will help us understand the severity of your migraine and the impact of migraine on your daily life.

**Please note:** These questions have all gone through a process called “validation.” They have been used in previous research with people who have migraine using the exact same language. This means that the research team who created the survey you are taking now were not able to change any of the words in the questions. Therefore, some of the language may not be words or descriptions that you use. We appreciate your participation.

***VALIDATED SCALE: MSQ v2.1***

**ASK EVERYONE**

**ALL QUESTIONS IN A1 SHOULD BE MANDATORY**

THE COA TITLE TO BE DISPLAYED IN BOLD AND CENTERED**:**

**MIGRAINE SPECIFIC QUALITY OF LIFE QUESTIONNAIRE (VERSION 2.1)**

**THE COPYRIGHT TO BE DISPLAYED ON THE BOTTOM LEFT-HAND CORNER OF THE TITLE SCREEN.**

Copyright 1992, 1996, 1998 Glaxo Wellcome Inc.

**DISPLAY BELOW LANGUAGE IN BOLD ON SEPARATE PAGE:**

**PATIENT INSTRUCTIONS:**

**Please fill out this questionnaire. It will help us understand the effects of migraine headache on your daily activities.**

**The questionnaire has been designed so that it can be completed quickly and easily. Please select only one answer for each question. You should answer every question.**

**Thank you for your time.**

**FOR ALL SCREENS IN A1 AFTER THE PATIENT INSTRUCTIONS, ADD A “BACK” BUTTON**

**FOR ALL A1 QUESTIONS BELOW, REMOVE “SELECT ONE” TEXT**

A1.	While answering the following questions, please think about ***all migraine attacks*** you may have	had ***in the past 4 weeks***.

1. In the past 4 weeks, how often have migraines **interfered** with how well you dealt with family, friends and others who are close to you?

| **None of the time** | **A little bit of the time** | **Some of the time** | **A good bit of the time** | **Most of the time** | **All of the time** |
| --- | --- | --- | --- | --- | --- |
| 6 | 5 | 4 | 3 | 2 | 1 |

2. In the past 4 weeks, how often have migraines **interfered** with your leisure time activities such as reading or exercising?

| **None of the time** | **A little bit of the time** | **Some of the time** | **A good bit of the time** | **Most of the time** | **All of the time** |
| --- | --- | --- | --- | --- | --- |
| 6 | 5 | 4 | 3 | 2 | 1 |

3. In the past 4 weeks, how often have you had **difficulty** in performing work or daily activities because of migraine symptoms?

| **None of the time** | **A little bit of the time** | **Some of the time** | **A good bit of the time** | **Most of the time** | **All of the time** |
| --- | --- | --- | --- | --- | --- |
| 6 | 5 | 4 | 3 | 2 | 1 |

4. In the past 4 weeks, how often did migraines **keep you** from getting as much done at work or at home?

| **None of the time** | **A little bit of the time** | **Some of the time** | **A good bit of the time** | **Most of the time** | **All of the time** |
| --- | --- | --- | --- | --- | --- |
| 6 | 5 | 4 | 3 | 2 | 1 |

5. In the past 4 weeks, how often did migraines **limit** your ability to work or do other activities as carefully as you usually do them?

| **None of the time** | **A little bit of the time** | **Some of the time** | **A good bit of the time** | **Most of the time** | **All of the time** |
| --- | --- | --- | --- | --- | --- |
| 6 | 5 | 4 | 3 | 2 | 1 |

6. In the past 4 weeks, how often have migraines **left you too tired** to do work or daily activities?

| **None of the time** | **A little bit of the time** | **Some of the time** | **A good bit of the time** | **Most of the time** | **All of the time** |
| --- | --- | --- | --- | --- | --- |
| 6 | 5 | 4 | 3 | 2 | 1 |

7. In the past 4 weeks, how often have migraines **limited** the number of days you have felt energetic?

| **None of the time** | **A little bit of the time** | **Some of the time** | **A good bit of the time** | **Most of the time** | **All of the time** |
| --- | --- | --- | --- | --- | --- |
| 6 | 5 | 4 | 3 | 2 | 1 |

8. In the past 4 weeks, how often have you had to **cancel** work or daily activities because you had a migraine?

| **None of the time** | **A little bit of the time** | **Some of the time** | **A good bit of the time** | **Most of the time** | **All of the time** |
| --- | --- | --- | --- | --- | --- |
| 6 | 5 | 4 | 3 | 2 | 1 |

9. In the past 4 weeks, how often did you **need help** in handling routine tasks such as everyday household chores, doing necessary business, shopping, or caring for others when you had a migraine?

| **None of the time** | **A little bit of the time** | **Some of the time** | **A good bit of the time** | **Most of the time** | **All of the time** |
| --- | --- | --- | --- | --- | --- |
| 6 | 5 | 4 | 3 | 2 | 1 |

10. In the past 4 weeks, how often did you have to **stop** work or daily activities to deal with migraine symptoms?

| **None of the time** | **A little bit of the time** | **Some of the time** | **A good bit of the time** | **Most of the time** | **All of the time** |
| --- | --- | --- | --- | --- | --- |
| 6 | 5 | 4 | 3 | 2 | 1 |

11. In the past 4 weeks, how often were you **not able to go** to social activities such as parties, dinner with friends, because you had a migraine?

| **None of the time** | **A little bit of the time** | **Some of the time** | **A good bit of the time** | **Most of the time** | **All of the time** |
| --- | --- | --- | --- | --- | --- |
| 6 | 5 | 4 | 3 | 2 | 1 |

12. In the past 4 weeks, how often have you felt fed up or frustrated because of your migraines?

| **None of the time** | **A little bit of the time** | **Some of the time** | **A good bit of the time** | **Most of the time** | **All of the time** |
| --- | --- | --- | --- | --- | --- |
| 6 | 5 | 4 | 3 | 2 | 1 |

13. In the past 4 weeks, how often have you **felt** like you were a burden on others because of your migraines?

| **None of the time** | **A little bit of the time** | **Some of the time** | **A good bit of the time** | **Most of the time** | **All of the time** |
| --- | --- | --- | --- | --- | --- |
| 6 | 5 | 4 | 3 | 2 | 1 |

14. In the past 4 weeks, how often have you been **afraid** of letting others down because of your migraines?

| **None of the time** | **A little bit of the time** | **Some of the time** | **A good bit of the time** | **Most of the time** | **All of the time** |
| --- | --- | --- | --- | --- | --- |
| 6 | 5 | 4 | 3 | 2 | 1 |

**THE LINK BELOW SHOULD NOT BE A HYPERLINK. KEEP AS TEXT ONLY**

For any information on the use of the MSQ 2.1, please contact Mapi Research Trust, Lyon, France.

Internet: [<https://eprovide.mapi-trust.org>](https://eprovide.mapi-trust.org)

**NEXT SCREEN, SEPARATE PAGE CONTAINING A BACK BUTTON ON THE BOTTOM LEFT OF THE SCREEN AND A SUBMIT BUTTON ON THE BOTTOM RIGHT OF THE SCREEN.**

**You have provided a response to all questionnaire items.**

**If you would like to change your response tap Back. Tap Submit if you are ready to save your responses.**

***END OF VALIDATED SCALE***

***VALIDATED SCALE: MIBS-4***

**ASK EVERYONE**

A2.	Please answer each of the following statements about the effect of your headaches in the past 4 weeks on days when you are not having an attack. **(select one for each statement)**

Between headache attacks or at times when I do not have a headache:

| **SELECT ONE RESPONSE FOR EACH ROW** | | **Don’t know/NA** | **Never** | **Rarely** | **Some of the time** | **Much of the time** | **Most or all of the time** |
| --- | --- | --- | --- | --- | --- | --- | --- |
| 1 | My headaches affect my work or school at times when I do not have a headache | 0 | 1 | 2 | 3 | 4 | 5 |
| 2 | I worry about planning social or leisure activities because I might have a headache | 0 | 1 | 2 | 3 | 4 | 5 |
| 3 | My headaches impact my life at times when I do not have a headache | 0 | 1 | 2 | 3 | 4 | 5 |
| 4 | At times when I do not have a headache, I feel helpless because of my headaches | 0 | 1 | 2 | 3 | 4 | 5 |

***END OF VALIDATED SCALE***

***VALIDATED SCALE: PHQ-2 and GAD-2***

**ASK EVERYONE**

A3.	Over the last 2 weeks, how often have you been bothered by the following problems?

| **SELECT ONE RESPONSE FOR EACH** | | **Not at all** | **Several days** | **More than half the days** | **Nearly every day** |
| --- | --- | --- | --- | --- | --- |
| a | Feeling nervous, anxious or on edge | 0 | 1 | 2 | 3 |
| b | Not being able to stop or control worrying | 0 | 1 | 2 | 3 |
| c | Little interest or pleasure in doing things | 0 | 1 | 2 | 3 |
| d | Feeling down, depressed, or hopeless | 0 | 1 | 2 | 3 |

***END OF VALIDATED SCALE***

***VALIDATED SCALE: WPAI***

**ASK EVERYONE**

A4.	The following questions ask about the effect of your MIGRAINE on your ability to work and	perform regular activities.

1. Are you currently employed (working for pay)?

| **SELECT ONE** | | |
| --- | --- | --- |
| 1 | Yes |  |
| 2 | No | **SKIP TO A4_6** |

The next questions are about the **past seven days**, not including today.

2. During the past seven days, how many hours did you miss from work because of problems associated with your MIGRAINE?  *Include hours you missed on sick days, times you went in late, left early, etc., because of your MIGRAINE.  Do not include time you missed to participate in this study.*

**ENTER WHOLE NUMBERS; RANGE FOR EACH: 0-90**

_____ Hours

3. During the past seven days, how many hours did you miss from work because of any other reason, such as vacation, holidays, time off to participate in this study?

**ENTER WHOLE NUMBERS; RANGE FOR EACH: 0-90**

_____ Hours

4. During the past seven days, how many hours did you actually work?

**ENTER WHOLE NUMBERS; RANGE FOR EACH: 0-90**

_____ Hours

**IF 0, SKIP TO A4_6**

5. During the past seven days, how much did your MIGRAINE affect your productivity while you were working?

*Think about days you were limited in the amount or kind of work you could do, days you accomplished less than you would like, or days you could not do your work as carefully as usual.  If MIGRAINE affected your work only a little, choose a low number.  Choose a high number if MIGRAINE affected your work a great deal.*

Consider only how much MIGRAINE affected

productivity while you were working.

| **MIGRAINE had no effect on my work** |  | | | | | | | | | | | **MIGRAINE completely prevented me from working** |
| --- | --- | --- | --- | --- | --- | --- | --- | --- | --- | --- | --- | --- |
|  | 0 | 1 | 2 | 3 | 4 | 5 | 6 | 7 | 8 | 9 | 10 |  |

SELECT A NUMBER

6. During the past seven days, how much did your MIGRAINE affect your ability to do your regular daily activities, other than work at a job?

*By regular activities, we mean the usual activities you do, such as work around the house, shopping, childcare, exercising, studying, etc.  Think about times you were limited in the amount or kind of activities you could do and times you accomplished less than you would like.  If MIGRAINE affected your activities only a little, choose a low number.  Choose a high number if MIGRAINE affected your activities a great deal.*

Consider only how much MIGRAINE affected your ability

to do your regular daily activities, other than work at a job.

| **MIGRAINE had no effect on my daily activities** |  | | | | | | | | | | | **MIGRAINE completely prevented me from doing my daily activities** |
| --- | --- | --- | --- | --- | --- | --- | --- | --- | --- | --- | --- | --- |
|  | 0 | 1 | 2 | 3 | 4 | 5 | 6 | 7 | 8 | 9 | 10 |  |

SELECT A NUMBER

**SHOW AFTER A4_6. ON THE SAME PAGE AT THE BOTOM OF THE PAGE:**

*Reilly MC, Zbrozek AS, Dukes EM. The validity and reproducibility of a work productivity and activity impairment instrument. PharmacoEconomics 1993; 4(5):353-65.*

***END OF VALIDATED SCALE***

***VALIDATED SCALE: TSQM-9***

**ASK EVERYONE**

A5.	Please take some time to think about your level of satisfaction or dissatisfaction with Nurtec^®^ ODT (rimegepant). We are interested in your evaluation of the effectiveness and convenience of the medication *over the last two to three weeks*, *or since you last used it.* For each question, please select the response that most closely corresponds to your own experiences.

1. How satisfied or dissatisfied are you with the ability of the medication to prevent or treat your condition?

| **Extremely Dissatisfied** | **Very Dissatisfied** | **Dissatisfied** | **Somewhat Satisfied** | **Satisfied** | **Very Satisfied** | **Extremely Satisfied** |
| --- | --- | --- | --- | --- | --- | --- |
| 1 | 2 | 3 | 4 | 5 | 6 | 7 |

2. How satisfied or dissatisfied are you with the way the medication relieves your symptoms?

| **Extremely Dissatisfied** | **Very Dissatisfied** | **Dissatisfied** | **Somewhat Satisfied** | **Satisfied** | **Very Satisfied** | **Extremely Satisfied** |
| --- | --- | --- | --- | --- | --- | --- |
| 1 | 2 | 3 | 4 | 5 | 6 | 7 |

3. How satisfied or dissatisfied are you with the amount of time it takes the medication to start working?

| **Extremely Dissatisfied** | **Very Dissatisfied** | **Dissatisfied** | **Somewhat Satisfied** | **Satisfied** | **Very Satisfied** | **Extremely Satisfied** |
| --- | --- | --- | --- | --- | --- | --- |
| 1 | 2 | 3 | 4 | 5 | 6 | 7 |

4. How easy or difficult is it to use the medication in its current form?

| **Extremely Difficult** | **Very Difficult** | **Difficult** | **Somewhat Easy** | **Easy** | **Very Easy** | **Extremely Easy** |
| --- | --- | --- | --- | --- | --- | --- |
| 1 | 2 | 3 | 4 | 5 | 6 | 7 |

5. How easy or difficult is it to plan when you will use the medication each time?

| **Extremely Difficult** | **Very Difficult** | **Difficult** | **Somewhat Easy** | **Easy** | **Very Easy** | **Extremely Easy** |
| --- | --- | --- | --- | --- | --- | --- |
| 1 | 2 | 3 | 4 | 5 | 6 | 7 |

6. How convenient or inconvenient is it to take the medication as instructed?

| **Extremely Inconvenient** | **Very Inconvenient** | **Inconvenient** | **Somewhat Convenient** | **Convenient** | **Very Convenient** | **Extremely Convenient** |
| --- | --- | --- | --- | --- | --- | --- |
| 1 | 2 | 3 | 4 | 5 | 6 | 7 |

7. Overall, how confident are you that taking this medication is a good thing for you?

| **Not at All Confident** | **A Little Confident** | **Somewhat Confident** | **Very Confident** | **Extremely Confident** |
| --- | --- | --- | --- | --- |
| 1 | 2 | 3 | 4 | 5 |

8. How certain are you that the good things about your medication outweigh the bad things?

| **Not at All Certain** | **A Little Certain** | **Somewhat Certain** | **Very Certain** | **Extremely Certain** |
| --- | --- | --- | --- | --- |
| 1 | 2 | 3 | 4 | 5 |

9. Taking all things into account, how satisfied or dissatisfied are you with this medication?

| **Extremely Dissatisfied** | **Very Dissatisfied** | **Dissatisfied** | **Somewhat Satisfied** | **Satisfied** | **Very Satisfied** | **Extremely Satisfied** |
| --- | --- | --- | --- | --- | --- | --- |
| 1 | 2 | 3 | 4 | 5 | 6 | 7 |

***END OF VALIDATED SCALE***

***VALIDATED SCALE: mTOQ-4***

**IF ACUTE ASK 1-4**

**IF LONG-TERM PREVENTION OR SHORT-TERM PREVENTION ASK 2-3**

A6.

1. After taking your migraine medication, are you pain-free within 2 hours for most attacks?

| **Never** | **Rarely** | **Less than half the time** | **Half the time or greater** |
| --- | --- | --- | --- |
| 0 | 0 | 1 | 2 |

2. Does 1 dose of your migraine medication usually relieve your headache and keep it away for at least 24 hours?

| **Never** | **Rarely** | **Less than half the time** | **Half the time or greater** |
| --- | --- | --- | --- |
| 0 | 0 | 1 | 2 |

3. Are you comfortable enough with your migraine medication to be able to plan your daily activities?

| **Never** | **Rarely** | **Less than half the time** | **Half the time or greater** |
| --- | --- | --- | --- |
| 0 | 0 | 1 | 2 |

4. After taking your migraine medication, do you feel in control of your migraines enough so that you feel you can return to normal function?

| **Never** | **Rarely** | **Less than half the time** | **Half the time or greater** |
| --- | --- | --- | --- |
| 0 | 0 | 1 | 2 |

**Programmer: Add up the above scores**

**(0) very poor treatment efficacy, (1-5) poor treatment efficacy, (6-7) moderate treatment efficacy, (8) maximum treatment efficacy1**

***END OF VALIDATED SCALE***

**End Section A**

**Section B: Medication Details**

**ASK EVERYONE**

B1a.	How many days in **the past 30 days** have you had a headache of any kind? If a headache lasted more than one day, please count the number of days that you experienced this headache.

*If you are unsure, please provide your best estimate.* **NUMERIC**

|_|_| **RANGE 0-30**

99 Don’t know

**IF B1a >0 ASK**

B1b.	Of the **[INSERT NUMBER FROM B1A]** headaches you have had in **the past 30 days**, how many of those have been migraine attacks?

|_|_| **RANGE 0-B1A RESPONSE**

99 Don’t know

**HIDE B1_2, 3, 4,5, 7 IF S3=99**

B1.	**[IF ACUTE:** In addition to Nurtec^®^ ODT (rimegepant)**]**, do you **currently** use any of the following for **acute treatment HOVER FOR DEFINITION OF ACUTE**?

**HOVER FOR DEFINITION: ACUTE**: The goal is to stop, relieve, or lower symptoms when **a migraine attack occurs**.

| **SELECT ALL THAT APPLY** | |
| --- | --- |
| 1 | Over-the-counter medication |
| 2 | Prescription oral medication |
| 3 | Prescription self-injection medication |
| 4 | Prescription nasal spray |
| 5 | Prescription topical gel or cream |
| 6 | A medical device to relieve, stop, or lower symptoms during a migraine attack (such as an external stimulator device) |
| 7 | Injections from my healthcare provider (such as nerve blocks) |
| 8 | Home remedies (e.g., ice pack, heat pad, yoga, etc.) |
| 99 | I do not use any products **[IF ACUTE:** besides Nurtec^®^ ODT (rimegepant)**]** to stop, relieve or lower symptoms **when a migraine attack occurs** or when I think it is about to occur |

**ASK B2 IF B1=1- 8; INSERT ONLY THOSE SELECTED IN B1**

B2.	Please think about **all** the treatments you **currently** use for **acute treatment** of a migraine attack **[IF ACUTE:** in addition to Nurtec^®^ ODT (rimegepant))**]**.

Please enter the number of each **different** type of treatment(s) you use currently.

| **ENTER WHOLE NUMBERS; RANGE FOR EACH: 1-120** | | |
| --- | --- | --- |
| 1 | Over-the-counter medication | _____ |
| 2 | Prescription oral medication | _____ |
| 3 | Prescription self-injection medication | _____ |
| 4 | Prescription nasal spray | _____ |
| 5 | Prescription topical gel or cream | _____ |
| 6 | A medical device to relieve, stop, or lower symptoms during a migraine attack (such as an external stimulator device | _____ |
| 7 | Injections from my healthcare provider (such as nerve blocks) | _____ |
| 8 | Home remedies (e.g., ice pack, heat pad, yoga, etc.) | _____ |

**HIDE B3_2, 6, 7, 8 IF S4 = 99**

B3.	**[IF LTP, STP:** In addition **to** Nurtec^®^ ODT (rimegepant)**]**, Do you **currently** use any of the following to **prevent migraine attacks from occurring**?

| **SELECT ALL THAT APPLY** | |
| --- | --- |
| 1 | Over-the-counter medication |
| 2 | Prescription oral medication |
| 3 | Behavioral treatments for preventing migraine attacks |
| 4 | Surgery to prevent or reduce migraine attacks |
| 5 | A device to prevent or reduce migraine attacks (such as an implant or external stimulator device) |
| 6 | Injections from my healthcare provider (such as Botox) |
| 7 | Prescription self-injection medication |
| 8 | Infusion |
| 9 | Home remedies (e.g., massages, chiropractor, yoga, etc.) |
| 99 | I do not use any products **[IF LTP, STP:** besides Nurtec^®^ ODT (rimegepant)**]** to prevent migraine attacks from occurring **[EXCLUSIVE]** |

**ASK B3a IF B3 = 6, 7, 8**

B3a.	Do you experience a “wearing off” effect between doses of your current injection or infusion treatment (i.e. your migraine attacks return or worsen before your next injection or infusion is due to be taken)?

| **SELECT ONE** | |
| --- | --- |
| 1 | Yes |
| 2 | No |
| 99 | Unsure |

**ASK B3b IF B3a = 1 YES**

B3b.	Do you use Nurtec^®^ ODT (rimegepant) to prevent migraine attacks in between your injections, as the effect of the previous injection wears off?

| **SELECT ONE** | |
| --- | --- |
| 1 | Yes |
| 2 | No |
| 99 | Unsure |

**ASK B4 IF B3=1-9; INSERT ONLY THOSE SELECTED IN B3**

B4.	Please think about **all** the treatment(s) you **currently** use **to prevent migraine attacks** **[IF LTP, STP:** addition to Nurtec^®^ ODT (rimegepant)**]**.

Please enter the number of each **different** type of treatment(s) you use currently.

| **ENTER WHOLE NUMBERS; RANGE FOR EACH: 1-20** | | |
| --- | --- | --- |
| 1 | Over-the-counter medication | _____ |
| 2 | Prescription oral medication | _____ |
| 3 | Behavioral treatments for preventing migraine attacks | _____ |
| 4 | Surgery to prevent or reduce migraine attacks | _____ |
| 5 | A device to prevent or reduce migraine attacks (such as an implant or external stimulator device) | _____ |
| 6 | Injections from my healthcare provider (such as Botox) | _____ |
| 7 | Prescription self-injection medication | _____ |
| 8 | Infusion | _____ |
| 8 | Home remedies (e.g., massages, chiropractor, yoga, etc.) | _____ |

B5.	Which of the following have you **previously used** to stop, relieve, or lower symptoms **when a migraine attack occurs?**

Please do not consider the treatments you currently use or plan to use.

| **SELECT ALL THAT APPLY; INSERT THOSE NOT SELECTED IN B1** | |
| --- | --- |
| 1 | Over-the-counter medication |
| 2 | Prescription oral medication |
| 3 | Prescription self-injection medication |
| 4 | Prescription nasal spray |
| 5 | Prescription topical gel or cream |
| 6 | A medical device to relieve, stop, or lower symptoms during a migraine attack (such as an external stimulator device) |
| 7 | Injections from my healthcare provider (such as nerve blocks) |
| 8 | Home remedies (e.g., ice pack, heat pad, yoga, etc.) |
| 99 | I have never used any of these **[EXCLUSIVE]** to stop, relieve or lower symptoms **when a migraine attack occurs** or when I think it is about to occur |

B6.	**IF LTP, STP: Earlier, you mentioned you currently take the following prescription medications to prevent migraine attacks: [PIPE IN S4 SELECTIONS HERE).**

Did you previously take any other prescription medications **to** **prevent migraine attacks**?

**IF ACUTE ONLY:** Have you ever taken any prescription medications to prevent migraine attacks?

| **SELECT ONE** | |
| --- | --- |
| 1 | Yes |
| 2 | No |

**IF B6 = 1 ASK B7**

B7.	Which prescription medications did you previously take to **prevent migraine attacks**?

| **SELECT ALL THAT APPLY** | |
| --- | --- |
| 1 | Aimovig^®^ (erenumab) |
| 2 | Ajovy^®^ (fremanezumab) |
| 3 | Botox^®^ (onabotulinumtoxinA) |
| 4 | Elavil^®^ (amitriptyline) |
| 5 | Emgality^®^ (galcanezumab) |
| 6 | Inderal^®^ (propranalol) |
| 7 | Nurtec^®^ ODT (rimegepant) |
| 8 | Qulipta^®^ (atogepant) |
| 9 | Topamax^®^ (topiramate) |
| 10 | Vyepti^®^ (eptinezumab) |
| 97 | Other prescription medications |
| 99 | I have not used any prescription medications to **prevent migraine attacks from occurring [EXCLUSIVE]** |

B8.	Please select how much you agree with the following:

| **SELECT AT LEAST ONE PER ROW.** | | **Almost always** | **Often** | **Sometimes** | **Rarely** | **Never** |
| --- | --- | --- | --- | --- | --- | --- |
| 1 | Nurtec^®^ ODT (rimegepant) will prevent me from having a migraine attack **[ASK IF LTP, STP]** | 1 | 2 | 3 | 4 | 5 |
| 2 | Nurtec^®^ ODT (rimegepant) will stop, relieve or lower my symptoms when a migraine attack occurs or when I think it is about to occur **[ASK IF ACUTE]** | 1 | 2 | 3 | 4 | 5 |

***END SECTION B***

**Section C: Long-Term Prevention**

**SHOW INTRO AND C1-C2 TO LONG-TERM PREVENTION USERS**

**SHOW INTRO ON SEPARATE PAGE**

**INTRODUCTION:**

You previously mentioned that you take Nurtec^®^ ODT ODT (rimegepant) consistently/regularly (every other day) without breaks to prevent or reduce the severity or frequency of migraine attacks.

In the following section, we are going to refer to this as **Long-Term Prevention treatment.**

C1.	When was the **first** time you took Nurtec^®^ ODT (rimegepant) as a **Long-Term Prevention treatment**? **[HOVER FOR DEFINITION OF LTP]**?

**[HOVER DEFINITION: LONG-TERM PREVENTION:** To prevent or reduce the severity or frequency of migraine attacks by taking consistently/regularly (every other day) without breaks.**]**

Please make your best guess if you are unsure.

**SHOW DROP-DOWN FOR “MONTH” AND “YEAR”.**

**MONTH + YEAR MUST BE EQUAL TO OR MORE RECENT THAN S5**

C1a.	How many times in the last **30 days** have you taken Nurtec^®^ ODT (rimegepant as a **Long-Term Prevention treatment**? **[HOVER FOR DEFINITION OF LTP]**?

**[HOVER DEFINITION: LONG-TERM PREVENTION:** To prevent or reduce the severity or frequency of migraine attacks by taking consistently/regularly (every other day) without breaks.**]**

Please make your best guess if you are unsure. Think only about times how often you took Nurtec ODT (rimegepant) to prevent a migraine attack, not when you wanted to stop one that was already occurring.

___ times **RANGE=0-18**

**FLAG IF <12 ENTERED**

**IF C1a = 0, SHOW ERROR MESSAGE “You mentioned earlier that you had taken Nurtec ODT (rimegepant) in the past 30 days. Please review these questions again.”**

**IF C1A = 0 ASK C1C**

C1b.	How many times in the last **3 months** have you taken Nurtec^®^ ODT (rimegepant) as a **Long-Term Prevention** treatment? **[HOVER FOR DEFINITION OF LTP]**

**[HOVER DEFINITION: LONG-TERM PREVENTION:** To prevent or reduce the severity or frequency of migraine attacks by taking consistently/regularly (every other day) without breaks.**]**

Please make your best guess if you are unsure. Think only about times you took Nurtec^®^ ODT (rimegepant) to prevent a migraine attack, not when you were already experiencing one.

___ times **RANGE=0-54**

C1c.	How easy or difficult is it to take Nurtec^®^ ODT (rimegepant) every other day?

| **SELECT ONE PER ROW** | | | | |
| --- | --- | --- | --- | --- |
| **Very Easy** | **Easy** | **Neither Easy nor Difficult** | **Difficult** | **Very Difficult** |
| 1 | 2 | 3 | 4 | 5 |

C2.	Why do you take Nurtec^®^ ODT (rimegepant) as a **Long-Term Prevention treatment**? **[HOVER FOR DEFINITION OF LTP]**?

**[HOVER DEFINITION: LONG-TERM PREVENTION:** To prevent or reduce the severity or frequency of migraine attacks by taking consistently/regularly (every other day) without breaks.**]**

| **SELECT ALL THAT APPLY, RANDOMIZE** | |
| --- | --- |
| 1 | It reduces the frequency of my migraine attacks |
| 2 | It reduces the severity of my migraine attacks |
| 3 | I am not bothered by side effects |
| 4 | It is affordable |
| 5 | It is covered by my insurance |
| 6 | I can take it with other medications |
| 7 | My doctor recommended it |
| 8 | It is convenient |
| 9 | I have frequent migraine attacks |
| 10 | I have severe migraine attacks |
| 11 | I have tried several other medications and was not satisfied |
| 97 | Another reason **ANCHOR** |

***END SECTION C***

**Section D: Short-Term Prevention**

**IF USE NURTEC** **ODT (RIMEGEPANT) AS SHORT-TERM PREVENTION (S7=2)**

**SHOW INTRO ON SEPARATE PAGE**

**INTRODUCTION:**

You previously mentioned that you take Nurtec^®^ ODT (rimegepant) for a limited period of time before a known migraine trigger **HOVER FOR MIGRAINE TRIGGER DEFINITION** or a specific situation when they want to reduce the chances of having a migraine attack. This is called **SHORT-TERM PREVENTION** treatment.

**HOVER FOR DEFINITION**: Migraine Trigger: An external or internal factor (e.g., stress, poor sleep quality, diet, dehydration and/or alcohol/caffeine, etc.) that can cause a migraine attack either by itself or in combination with other factors.

**SHOW SHORT-TERM PREVENTION DIAGRAM FROM QUESTION S7**

In the following section, we are going to refer to this as **Short-Term Prevention treatment.**

D1.	When was the **first** time you took Nurtec^®^ ODT (rimegepant) as a **Short-Term Prevention** treatment? **[HOVER FOR DEFINITION OF STP]**

**[HOVER FOR DEFINITION: SHORT-TERM PREVENTION**: To prevent migraine attacks by taking medication for a limited period of time before a known **migraine trigger** or a specific situation when you want to reduce the chances of having a migraine attack.**]**

Please make your best guess if you are unsure. Think only about times you took Nurtec^®^ ODT (rimegepant) to prevent a migraine attack, not when you were already experiencing one.

**SHOW DROP-DOWN FOR “MONTH” AND “YEAR”.**

**MONTH + YEAR MUST BE EQUAL TO OR MORE RECENT THAN S5**

**IF ANSWERED, FLAG IN DATA FOR REPORTING**

D2.	How many times in the last **30 days** have you taken Nurtec^®^ ODT (rimegepant) as a **Short-Term Prevention** treatment? **[HOVER FOR DEFINITION OF STP]**

**[HOVER FOR DEFINITION: SHORT-TERM PREVENTION**: To prevent migraine attacks by taking medication for a limited period of time before a known **migraine trigger** or a specific situation when you want to reduce the chances of having a migraine attack.**]**

Please make your best guess if you are unsure. Think only about times you took Nurtec^®^ ODT (rimegepant) to prevent a migraine attack, not when you were already experiencing one.

___ times **RANGE=0-18**

**IF ANSWERED, FLAG IN DATA FOR REPORTING**

**IF D2 = 0 ASK D2A**

D2a.	How many times in the last **3 months** have you taken Nurtec^®^ ODT (rimegepant) as a **Short-Term Prevention** treatment? **[HOVER FOR DEFINITION OF STP]**

**[HOVER FOR DEFINITION: SHORT-TERM PREVENTION**: To prevent migraine attacks by taking medication for a limited period of time before a known **migraine trigger** or a specific situation when you want to reduce the chances of having a migraine attack.**]**

Please make your best guess if you are unsure. Think only about times you took Nurtec^®^ ODT (rimegepant) to prevent a migraine attack, not when you were already experiencing one.

___ times **RANGE=0-54**

**IF ANSWERED, FLAG IN DATA FOR REPORTING**

D3.	Why do you take Nurtec^®^ ODT (rimegepant) as a **Short-Term Prevention** treatment? **[HOVER FOR DEFINITION OF STP]**

**[HOVER FOR DEFINITION: SHORT-TERM PREVENTION**: To prevent migraine attacks by taking medication for a limited period of time before a known **migraine trigger** or a specific situation when you want to reduce the chances of having a migraine attack.**]**

| **SELECT ALL THAT APPLY, RANDOMIZE** | |
| --- | --- |
| 1 | It reduces the frequency of my migraine attacks |
| 2 | It reduces the severity of my migraine attacks |
| 3 | I am not bothered by side effects |
| 4 | It is affordable |
| 5 | It is covered by my insurance |
| 6 | I can take it with other medications |
| 7 | My doctor recommended it |
| 8 | It is convenient |
| 9 | I have frequent migraine attacks |
| 10 | I have severe migraine attacks |
| 11 | It helps me to worry less about expected migraine triggers |
| 97 | Another reason **ANCHOR** |

D4.	You mentioned earlier you take Nurtec ODT (rimegepant) as **Short-Term Prevention** treatment. **[HOVER FOR DEFINITION OF STP]**

**[HOVER FOR DEFINITION: SHORT-TERM PREVENTION**: To prevent migraine attacks by taking medication for a limited period of time before a known **migraine trigger** or a specific situation when you want to reduce the chances of having a migraine attack.**]**

During which of the following situations do you sometimes take Nurtec^®^ ODT (rimegepant) as a **Short-Term Prevention** treatment to reduce the likelihood of migraine attacks?

| **SELECT ALL THAT APPLY, RANDOMIZE** | |
| --- | --- |
| 1 | During work or school |
| 2 | While I spend time with family or friends |
| 3 | During vacations, holidays, or leisure time |
| 4 | During physical activity / exercise |
| 5 | To take breaks from taking it every other day |
| 6 | After I have experienced a migraine attack to prevent another one |
| 97 | Another reason **ANCHOR** |

D5.	Which of the following, if any, are migraine triggers **HOVER FOR MIGRAINE TRIGGER DEFINITION** for which you have taken or plan to take Nurtec ODT (rimegepant) as a **Short-Term Prevention** treatment? **[HOVER FOR DEFINITION OF STP]**?

**HOVER FOR DEFINITION**: Migraine Trigger: An external or internal factor (e.g., stress, poor sleep quality, diet, dehydration and/or alcohol/caffeine, etc.) that can cause a migraine attack either by itself or in combination with other factors.

| **SELECT ALL THAT APPLY, RANDOMIZE** | |
| --- | --- |
| 1 | Period / menstrual cycle **SHOW IF S1 = ≤ 60 AND S1b = 2 FEMALE** |
| 2 | Hormonal change (such as before or during menopause, ovulation, or as a result of taking hormone-altering medication or birth control) |
| 3 | Stress |
| 4 | Alcohol |
| 5 | Changes in weather |
| 6 | Seasonal change |
| 7 | Dietary factors / Specific food |
| 8 | Disruptive sleep patterns / Lack of sleep |
| 9 | Light |
| 10 | Odors |
| 11 | Exercise |
| 12 | Smoking (i.e., when you smoke cigarettes, e-cigarettes, vapes, etc.) **[KEEP 12 and 13 TOGETHER IN RANDOMIZATION]** |
| 13 | Second-hand smoking (when others smoke around you) **[KEEP 12 and 13 TOGETHER IN RANDOMIZATION]** |
| 13 | Neck Pain |
| 14 | Sex |
| 15 | Caffeine |
| 16 | Dehydration |
| 17 | Medication overuse |
| 18 | Skipping meals |
| 19 | Travel/air travel |
| 20 | Altitude |
| 21 | Looking at screens (such as TVs, computer monitors or smartphones) |
| 22 | Noise |
| 97 | Another reason **ANCHOR** |
| 99 | I don’t have any reliable migraine triggers **ANCHOR [EXCLUSIVE]** |

**IF ANY OPTION 1-97 SELECTED IN D5, ASK D6**

D6.	What is the likelihood of each of these triggers causing a migraine attack?

**DISPLAY A GRID WITH ONLY THOSE SELECTED IN D5:**

| **SELECT ONE PER ROW** | | | | |
| --- | --- | --- | --- | --- |
| **Extremely Unlikely** | **Unlikely** | **Neither Likely nor Unlikely** | **Likely** | **Extremely Likely** |
| 1 | 2 | 3 | 4 | 5 |

D7.	Thinking about the last time you took Nurtec^®^ ODT (rimegepant) as a **Short-Term Prevention** treatment **[HOVER FOR DEFINITION OF STP]**, for how long did you take it before you stopped?

**[HOVER FOR DEFINITION: SHORT-TERM PREVENTION**: To prevent migraine attacks by taking medication for a limited period of time before a known **migraine trigger** or a specific situation when you want to reduce the chances of having a migraine attack.**]**

| **SELECT ONE** | |
| --- | --- |
| 1 | 1 day |
| 2 | 2-3 days |
| 3 | 4-10 days |
| 4 | More than 10 days |

D8.	When you have taken Nurtec^®^ ODT (rimegepant) as a **Short-Term Prevention** treatment, how have you taken it? **[HOVER FOR DEFINITION OF STP]**?

**[HOVER FOR DEFINITION: SHORT-TERM PREVENTION**: To prevent migraine attacks by taking medication for a limited period of time before a known **migraine trigger** or a specific situation when you want to reduce the chances of having a migraine attack.**]**

| **SELECT ALL THAT APPLY** | |
| --- | --- |
| 1 | **One time** (one tablet), before a known trigger or specific situation |
| 2 | **Every day** for more than one day before/during a known trigger or specific situation |
| 3 | **Every other day** before/during a known trigger or specific situation |

D8a.	How satisfied or dissatisfied are you with taking Nurtec ODT (rimegepant) as a **Short-Term Prevention** treatment **[INSERT ANSWERS FROM D8]**? **[HOVER FOR DEFINITION OF STP]**

**[HOVER FOR DEFINITION: SHORT-TERM PREVENTION**: To prevent migraine attacks by taking medication for a limited period of time before a known **migraine trigger** or a specific situation when you want to reduce the chances of having a migraine attack.**]**

**DISPLAY A GRID WITH ONLY THOSE SELECTED IN D8**

| **SELECT ONE PER ROW** | | | | | | |
| --- | --- | --- | --- | --- | --- | --- |
| **Extremely Dissatisfied** | **Very Dissatisfied** | **Dissatisfied** | **Somewhat Satisfied** | **Satisfied** | **Very Satisfied** | **Extremely Satisfied** |
| 1 | 2 | 3 | 4 | 5 | 6 | 7 |

D9.	How frequently do you have triggers or situations for which you take Nurtec^®^ ODT (rimegepant) as a **Short-Term Prevention** treatment? **[HOVER FOR DEFINITION OF STP]**

**[HOVER FOR DEFINITION: SHORT-TERM PREVENTION**: To prevent migraine attacks by taking medication for a limited period of time before a known **migraine trigger** or a specific situation when you want to reduce the chances of having a migraine attack.**]**

| **SELECT ONE** | |
| --- | --- |
| 1 | More than once a month |
| 2 | About once a month |
| 3 | About once every two months |
| 4 | 3-4 times a year |
| 5 | Less than 3 times a year |

D10.	How did you learn about taking Nurtec^®^ ODT (rimegepant) as a **Short-Term Prevention** treatment? **[HOVER FOR DEFINITION OF STP]**?

**[HOVER FOR DEFINITION: SHORT-TERM PREVENTION**: To prevent migraine attacks by taking medication for a limited period of time before a known **migraine trigger** or a specific situation when you want to reduce the chances of having a migraine attack.**]**

| **SELECT ALL THAT APPLY** | |
| --- | --- |
| 1 | Physician who prescribed Nurtec^®^ ODT (rimegepant) told me |
| 2 | Pharmacist |
| 3 | Other healthcare provider |
| 4 | Social media / Advertisement |
| 5 | Friend |
| 6 | Family member |
| 7 | I experimented or tried it on my own |
| 8 | Patient support group or social advocacy group |
| 97 | Other |

***END SECTION E***

**Section F: Acute-only Treatment Details**

**IF USE NURTEC** **ODT (RIMEGEPANT) AS ACUTE**

**SHOW INTRO ON SEPARATE PAGE**

**INTRODUCTION**

You previously mentioned that you take Nurtec^®^ ODT (rimegepant) to stop, relieve, or lower symptoms **when a migraine attack occurs**. (This is also known as acute treatment)

In the following section, we are going to refer to this as **ACUTE treatment.**

**ASK ACUTE**

F1.	When was the **first** time you took Nurtec^®^ ODT (rimegepant) as an **acute treatment** **[HOVER FOR DEFINITION OF ACUTE]**?

**[HOVER FOR DEFINITION: ACUTE**: The goal is to stop, relieve, or lower symptoms when **a migraine attack occurs**.**]**

Please make your best guess if you are unsure. Think only about times you took Nurtec ODT (rimegepant) to stop, relieve or lower symptoms of a migraine attack that you were already experiencing.

**SHOW DROP-DOWN FOR “MONTH” AND “YEAR”.**

**MONTH + YEAR MUST BE EQUAL TO OR MORE RECENT THAN S5**

F2.	How many times in the last **30 days** have you taken Nurtec^®^ ODT (rimegepant) as an **acute treatment** **[HOVER FOR DEFINITION OF ACUTE]** for migraine?

**[HOVER FOR DEFINITION: ACUTE**: The goal is to stop, relieve, or lower symptoms when **a migraine attack occurs**.**]**

Please make your best guess if you are unsure. Think only about times you took Nurtec^®^ ODT (rimegepant) to stop, relieve, or lower symptoms of a migraine attack that you were already experiencing.

___ times **RANGE=0-18**

**IF F2 = 0, SHOW ERROR MESSAGE “You mentioned earlier that you had taken Nurtec ODT (rimegepant) in the past 30 days. Please review these questions again.”**

**IF F2 = 0 ASK F2A**

F2a.	How many times in the last **3 months** have you taken Nurtec ODT (rimegepant) as an **ACUTE** treatment? **[HOVER FOR DEFINITION OF STP]**

**[HOVER FOR DEFINITION: ACUTE**: The goal is to stop, relieve, or lower symptoms when **a migraine attack occurs**.**]**

Please make your best guess if you are unsure. Think only about times you took Nurtec^®^ ODT (rimegepant) to prevent a migraine attack, not when you were already experiencing one.

___ times **RANGE=0-54**

F3.	You mentioned using Nurtec^®^ ODT (rimegepant) **acute treatment** **[HOVER FOR DEFINITION OF ACUTE]**. When do you usually take Nurtec ODT (rimegepant)?

**[HOVER FOR DEFINITION: ACUTE**: The goal is to stop, relieve, or lower symptoms when **a migraine attack occurs**.**]**

| **SELECT ALL THAT APPLY** | |
| --- | --- |
| 1 | At the first sign of an attack (but before pain starts) |
| 2 | When the pain is mild |
| 3 | When the pain is moderate |
| 4 | When the pain is severe |
| 5 | Only when I am doing a specific activity that would be negatively impacted by a migraine attack |

**END SECTION F**

**Section J: Non-Short-Term Prevention Users**

**SHOW IF S7** **≠ 2 SHORT-TERM PREVENTION**

J1.	There are some people who experience migraine attacks who take Nurtec^®^ ODT (rimegepant) for a limited period of time before a known migraine trigger **HOVER FOR MIGRAINE TRIGGER DEFINITION** or a specific situation when they want to reduce the chances of having a migraine attack. This is called **SHORT-TERM PREVENTION** treatment.

**HOVER FOR DEFINITION**: Migraine Trigger: An external or internal factor (e.g., stress, poor sleep quality, diet, dehydration and/or alcohol/caffeine, etc.) that can cause a migraine attack either by itself or in combination with other factors.

**SHOW SHORT-TERM PREVENTION DIAGRAM FROM QUESTION S7**

Would you be interested in taking Nurtec^®^ ODT (rimegepant) as a **Short-Term Prevention** treatment?

| **SELECT ONLY ONE** | |
| --- | --- |
| 1 | Yes |
| 2 | No |
| 3 | Don’t know |

J2.	Which of the following, if any, are migraine triggers **HOVER FOR MIGRAINE TRIGGER DEFINITION** for you?

**HOVER FOR DEFINITION**: Migraine Trigger: An external or internal factor (e.g., stress, poor sleep quality, diet, dehydration and/or alcohol/caffeine, etc.) that can cause a migraine attack either by itself or in combination with other factors.

| **SELECT ALL THAT APPLY, RANDOMIZE** | |
| --- | --- |
| 1 | Period / menstrual cycle **SHOW IF S1 = ≤ 60 AND S1b = 2 FEMALE** |
| 2 | Hormonal change (such as before or during menopause, ovulation, or as a result of taking hormone-altering medication or birth control) |
| 3 | Stress |
| 4 | Alcohol |
| 5 | Changes in weather |
| 6 | Seasonal change |
| 7 | Dietary factors / Specific food |
| 8 | Disruptive sleep patterns / Lack of sleep |
| 9 | Light |
| 10 | Odors |
| 11 | Exercise |
| 12 | Smoking (i.e., when you smoke cigarettes, e-cigarettes, vapes, etc.) **[KEEP 12 and 13 TOGETHER IN RANDOMIZATION]** |
| 13 | Second-hand smoking (when others smoke around you) **[KEEP 12 and 13 TOGETHER IN RANDOMIZATION]** |
| 14 | Neck Pain |
| 15 | Sex |
| 16 | Caffeine |
| 17 | Dehydration |
| 18 | Medication overuse |
| 19 | Skipping meals |
| 20 | Travel/air travel |
| 21 | Altitude |
| 22 | Looking at screens (such as TVs, computer monitors or smartphones) |
| 23 | Noise |
| 97 | Another reason **ANCHOR** |
| 99 | I don’t have any reliable migraine triggers **ANCHOR [EXCLUSIVE]** |

**IF J2=1-97 ASK J2a**

J2a.	Would you be interested in taking Nurtec^®^ ODT (rimegepant) to avoid a migraine attack(s) caused by any of the trigger(s) you selected?

| **SELECT ALL THAT APPLY** | |
| --- | --- |
| 1 | **PIPE IN ANSWES SELECTED IN J2** |
| 99 | I would not be interested in taking Nurtec^®^ ODT (rimegepant) to prevent migraine attacks associated with my triggers **ANCHOR [EXCLUSIVE]** |

**IF ANY OPTION 1-97 SELECTED IN J2, ASK J2b**

J2b.	What is the likelihood of each of these triggers causing a migraine attack?

**DISPLAY A GRID WITH ONLY THOSE SELECTED IN J2:**

| **SELECT ONE PER ROW** | | | | |
| --- | --- | --- | --- | --- |
| **Extremely Unlikely** | **Unlikely** | **Neither Likely nor Unlikely** | **Likely** | **Extremely Likely** |
| 1 | 2 | 3 | 4 | 5 |

**IF J1=1 OR 3 ASK J3**

J3.	What type of scenarios/situations would you be interested in taking Nurtec^®^ ODT (rimegepant) as a **Short-Term Prevention** treatment to avoid migraine attacks?

| **SELECT ALL THAT APPLY, RANDOMIZE** | |
| --- | --- |
| 1 | During work or school |
| 2 | While I spend time with family or friends |
| 3 | During vacations, holidays, or leisure time |
| 4 | During physical activity / exercise |
| 5 | To take breaks from taking it every other day without breaks (e.g., long-term prevention) |
| 6 | After I have experienced a migraine attack to prevent another one |
| 97 | Another reason **ANCHOR** |

**IF J1=1 OR 3, ASK J4**

J4.	Why are you not currently taking Nurtec^®^ ODT (rimegepant) as a **Short-Term Prevention** treatment?

| **SELECT ALL THAT APPLY, RANDOMIZE** | |
| --- | --- |
| 1 | I am afraid my migraine attacks won’t be as well managed |
| 2 | Decided not to after speaking with my doctor |
| 3 | I have not talked about it with my doctor |
| 4 | I am worried that it won’t work well for me |
| 5 | I am worried about side effects from stopping and starting Nurtec^®^ ODT (rimegepant) |
| 6 | I do not have enough Nurtec^®^ ODT (rimegepant) pills |
| 97 | Another reason **ANCHOR** |

**CANNOT SELECT BOTH 2 AND 3**

**IF J1=2, ASK J5**

J5.	Why would you not be interested in taking Nurtec^®^ ODT (rimegepant) as a **Short-Term Prevention** treatment?

| **SELECT ALL THAT APPLY, RANDOMIZE** | |
| --- | --- |
| 1 | I am afraid I will experience more migraine attacks |
| 2 | My doctor has not recommended it to me |
| 3 | I have not talked about it with my doctor |
| 4 | I am worried that it won’t work well for me |
| 5 | I am worried about side effects from stopping and starting Nurtec^®^ ODT (rimegepant) |
| 6 | I am happy with how I currently take Nurtec^®^ ODT (rimegepant) |
| 7 | I cannot predict when I would need to take it as a Short-Term Prevention |
| 8 | I am concerned my insurance will not cover the extra medication |
| 9 | I worry insurance wouldn’t cover the cost |
| 10 | I am concerned that my discount card would no longer work |
| 11 | **IF S7=1 ACUTE:** I am worried about the long-term effects of taking Nurtec ODT (rimegepant) |
| 97 | Another reason **ANCHOR** |

**CANNOT SELECT J5_2 AND J5_3**

J6.	Which of the following are you **likely** to do in the next few months?

When you answer, keep in mind that “**Short-Term Prevention** treatment” means taking Nurtec^®^ ODT (rimegepant) for a limited period of time before a known migraine trigger **HOVER FOR MIGRAINE TRIGGER DEFINITION** or a specific situation when you want to reduce the chances of having a migraine attack.

**HOVER FOR DEFINITION**: Migraine Trigger: An external or internal factor (e.g., stress, poor sleep quality, diet, dehydration and/or alcohol/caffeine, etc.) that can cause a migraine attack either by itself or in combination with other factors.

| **SELECT ALL THAT APPLY, RANDOMIZE** | |
| --- | --- |
| 1 | Try taking Nurtec^®^ ODT (rimegepant) as a Short-Term Prevention treatment |
| 2 | Talk to my doctor about taking Nurtec^®^ ODT (rimegepant) as a Short-Term Prevention treatment at my next scheduled appointment |
| 3 | Schedule an appointment to talk to my doctor about Nurtec^®^ ODT (rimegepant) as a Short-Term Prevention treatment |
| 4 | Do more research on my own about Nurtec^®^ ODT (rimegepant) as a Short-Term Prevention treatment |
| 5 | Talk to someone else I know who has migraine about Nurtec^®^ ODT (rimegepant) as a Short-Term Prevention treatment (either in person, over the phone, or over the internet) |
| 6 | Talk to my insurance company about coverage |
| 99 | None of the above **ANCHOR, EXCLUSIVE** |

**END SECTION**

**Section G: Nurtec** **ODT (rimegepant) Prescribing Details**

**ASK EVERYONE**

**SHOW G1-G2 ON SAME SCREEN**

G1.	Please think about when you first received a prescription for Nurtec^®^ ODT (rimegepant). During what type of appointment did this happen?

| **SELECT ONLY ONE** | |
| --- | --- |
| 1 | In-person appointment (e.g., at a doctor’s office) |
| 2 | Via telehealth / phone / video chat |
| 3 | Via messaging my doctor on my patient portal (I did not have an actual appointment) |
| 99 | Don’t recall |

G2.	Which type of healthcare provider first prescribed Nurtec^®^ ODT (rimegepant) to you?

| **SELECT ONLY ONE** | |
| --- | --- |
| 1 | Primary Care Provider |
| 2 | General Neurologist (not a headache specialist) |
| 3 | Headache Specialist |
| 4 | Pain Specialist |
| 5 | OB-GYN (Obstetrician or Gynecologist) |
| 6 | Emergency Room (ER) at a hospital |
| 7 | Urgent Care Center |
| 8 | Nurse Practitioner (NP) or Physician Assistant (PA) |
| 97 | Other type of Doctor or Specialist Office / Clinic |
| 99 | Don’t recall |

G3.	How did your doctor instruct you to take Nurtec ODT (rimegepant)?

| **SELECT ALL THAT APPLY** | |
| --- | --- |
| 1 | To treat to stop, relieve, or lower symptoms **when a migraine attack occurs**. (This is also known as acute treatment) |
| 2 | To prevent migraine attacks by taking medication for a limited period of time before a known migraine trigger or a specific situation when you want to reduce the chances of having a migraine attack. (This is also known as short-term prevention treatment) |
| 3 | To prevent or reduce the severity or frequency of migraine attacks by taking consistently/regularly (every other day) without breaks. (this is also known as long-term prevention) |
| 99 | Don’t recall **[EXCLUSIVE]** |

***END SECTION G***

**Section H: General Health and Clinical Characteristics**

**ASK EVERYONE:**

H1.	When did a healthcare provider **first** diagnose you with migraine? *Please make your best guess if you are unsure.*

**SHOW DROP-DOWN FOR “MONTH” AND “YEAR”.**

**MUST BE EARLIER OR EQUAL TO S5, MUST NOT BE EARLIER THAN YEAR OF BIRTH(YEAR OF BIRTH CAN BE ESTIMATE FROM S1a)**

H1a.	When did you first experience migraine attacks (**before** **diagnosis** by your healthcare provider)? *Please make your best guess if you are unsure.*

**SHOW DROP-DOWN FOR “MONTH” AND “YEAR”.**

**MUST BE EARLIER OR EQUAL TO S5, MUST NOT BE EARLIER THAN YEAR OF BIRTH(YEAR OF BIRTH CAN BE ESTIMATE FROM S1a), AND MUST BE LESS THAN H1**

H2a.	Height and weight may affect different aspects of your health or how well some treatments or medications may work.

What is your height in feet and inches?

Height (feet /inches)	______ft. ______ in. **[PROGRAMMER: CALCULATE TO INCHES, E.G., 5 FT + 5 IN = 65 IN] [RANGE FOR FT. 3-8; RANGE FOR IN. 0-11]**

H2b.	Please tell us your weight. If you don’t remember the exact number, please give us your best guess.

Weight (pounds)	______	**[PROGRAMMER: ALLOW WHOLE NUMBERS ONLY; RANGE 50-700 LBS]**

(99) Prefer not to answer

**Calculate height in inches = ft from S11 x 12 + inches from H2a**

**Calculate HIGHEST BMI = (weight in pounds from H2b x 703) / (H2A height in inches x H2A height in inches).**

H2c.	Which of the below have you been diagnosed with, or told that you have, by a healthcare provider?

| **SELECT ALL THAT APPLY** | |
| --- | --- |
| 1 | Insomnia |
| 2 | Depression |
| 3 | Anxiety |
| 4 | Gastric ulcer/GI bleeding |
| 5 | PAD |
| 6 | Angina |
| 7 | Allergies/hay fever |
| 8 | Epilepsy |
| 9 | Arthritis, type unknown |
| 10 | Stroke or TIA |
| 11 | Rheumatoid arthritis |
| 12 | Asthma |
| 13 | Vitamin D deficiency |
| 14 | Psoriasis |
| 15 | Osteoarthritis |
| 16 | Rosacea |
| 17 | Myocardial infarction |
| 18 | High cholesterol |
| 19 | Hypertension |
| 20 | Kidney disease |
| 21 | Diabetes |
| 98 | None of the above **[EXCLUSIVE]** |
| 99 | Prefer not to answer **[EXCLUSIVE]** |

H3.	Which of the following, if any, do you currently use?

| **SELECT ALL THAT APPLY** | |
| --- | --- |
| 1 | Cigarettes |
| 2 | Electronic cigarettes / vape |
| 3 | Nicotine replacements (e.g., nicotine gum, lozenges) |
| 4 | Smokeless tobacco products (e.g., dip, chewing tobacco, nicotine pouches) |
| 5 | Cigars |
| 98 | None of the above **[EXCLUSIVE]** |
| 99 | Prefer not to answer **[EXCLUSIVE]** |

H4.	How often do you drink alcohol?

| **SELECT ONLY ONE** | |
| --- | --- |
| 1 | Never |
| 2 | Once a month or less often |
| 3 | 2 to 4 times a month |
| 4 | 2 to 3 times a week |
| 5 | 4 or more times a week |
| 99 | Prefer not to answer |

**IF NOT “NEVER” (H4=2-99), ASK**

H4a.	How many drinks containing alcohol do you have on a typical day when you are drinking?

| **SELECT ONLY ONE** | |
| --- | --- |
| 1 | 1 or 2 |
| 2 | 3 or 4 |
| 3 | 5 or 6 |
| 4 | 7 to 9 |
| 5 | 10 or more |
| 99 | Prefer not to answer |

**Section I: Sociodemographics**

The last set of questions are for classification purposes only.

I2.	In what US state do you live?

**[DROP-DOWN FOR STATE]**

**PROGRAMMER: ESTABLISH 4 GEOGRAPHIC CENSUS REGIONS AND TRACK IN FIELD REPORT**

I3.	What is your current **marital status**?

| **SELECT ONE** |  |
| --- | --- |
| Single, never married | 1 |
| Committed relationship / Married | 2 |
| Separated / Divorced | 3 |
| Widowed | 4 |
| I prefer not to answer | 98 |

I4.	What is the highest level of formal **education** you have completed?

| **SELECT ONE** |  |
| --- | --- |
| Some high school or less | 1 |
| High school graduate or equivalent (e.g., GED) | 2 |
| Completed some college, but no degree | 3 |
| Associate degree (e.g., AA, AS, AAS) | 4 |
| College graduate (e.g., BA, AB, BS) | 5 |
| Completed some graduate school, but no degree | 6 |
| Completed graduate school (e.g., MS, MD, PhD) | 7 |
| I prefer not to answer | 98 |

**IF I4= 7 ASK I4a**

I4a.	What type of graduate degree do you have?

| **SELECT ALL THAT APPLY** |  |
| --- | --- |
| Master of Arts (MA), Master of Science (MSc) | 1 |
| Nursing (NP, RN) | 2 |
| Pharmacy (PharmD) | 3 |
| Law Degree (LLM, JD) | 4 |
| Medical Degree (MD, DO) | 5 |
| Doctoral Degree (PhD, ScD) | 6 |
| Other Degree | 97 |

I5.	What is your **employment status**?

| **SELECT ONE** |  |
| --- | --- |
| Employed full-time **HIDE IF A4_1=2** | 1 |
| Employed part-time **HIDE IF A4_1=2** | 1 |
| Employed, but currently on temporary leave of absence | 2 |
| Not employed **HIDE IF A4_1=1** | 4 |
| Student **HIDE IF A4_1=1** | 7 |
| Other | 97 |

I6.	What was your total annual **household** income before taxes for 2023? Please include money earned by you personally, your spouse / partner, and any other adults in the household. Please give us your best estimate if you’re not sure.

| **SELECT ONE** |  |
| --- | --- |
| Less than $15,000 | 1 |
| $15,001 to $30,000 | 2 |
| $30,001 to $60,000 | 3 |
| $60,001 to $90,000 | 4 |
| $90,001 to $150,000 | 5 |
| More than $150,000 | 9 |
| I prefer not to answer | 98 |

I7.	Which of the following best describes the location where you live?

| **SELECT ONLY ONE** | |
| --- | --- |
| 1 | Major metropolitan area, population > 500,000 |
| 2 | Urban area, population between 100,000 and 500,000 |
| 3 | Suburb of a large city, population > 100,000 |
| 4 | Small city, population between 30,000 and 100,000 |
| 5 | Rural or small town, population < 30,000 |

I8.	What kind of health insurance do you have currently?

Please select ALL the types of health insurance you have.

| **SELECT ALL THAT APPLY** | |
| --- | --- |
| 1 | Individual/Family insurance plans (i.e., private coverage purchased through your employer, your spouse’s employer, or directly by you) |
| 2 | Medicaid |
| 3 | Other (Including TRICARE and VA/Champus) |
| 4 | Other |
| 5 | Not Sure **EXCLUSIVE** |
| 6 | I do not have health insurance **EXCLUSIVE** |
| 98 | I prefer not to answer **EXCLUSIVE** |

I9.	What is your race?

| **SELECT ALL THAT APPLY** | |
| --- | --- |
| 1 | African American / Black |
| 2 | Asian |
| 3 | American Indian / Alaska Native |
| 4 | Native Hawaiian or Other Pacific Islander |
| 5 | White or Caucasian |
| 6 | Other |
| 98 | Prefer not to answer **EXCLUSIVE** |

I10.	Are you of Hispanic or Latino/a origin?

| **SELECT ONE** | |
| --- | --- |
| 1 | Yes, Hispanic or Latino/a origin |
| 2 | No, not Hispanic or Latino/a origin |
| 99 | Prefer not to answer |

I11a.	How often do you have someone help you read medical information materials?

| **Always** | **Often** | **Sometimes** | **Occasionally** | **Never** |
| --- | --- | --- | --- | --- |
| 1 | 2 | 3 | 4 | 5 |

I11b.	How confident are you filling out medical forms by yourself?

| **Extremely** | **Quite a bit** | **Somewhat** | **A little bit** | **Not at all** |
| --- | --- | --- | --- | --- |
| 1 | 2 | 3 | 4 | 5 |

I11c.	How often do you have problems learning about your medical condition because of difficulty understanding written information?

| **Always** | **Often** | **Sometimes** | **Occasionally** | **Never** |
| --- | --- | --- | --- | --- |
| 1 | 2 | 3 | 4 | 5 |

**SHOW ONCE PARTICIPANT HAS SUCCESFULLY COMPLTED SURVEY: “**You have reached the end of the survey. Thank you for your participation!”

**END OF SURVEY**
